# Mathematical model and tool to explore shorter multi-drug therapy options for active pulmonary tuberculosis

**DOI:** 10.1371/journal.pcbi.1008107

**Published:** 2020-08-18

**Authors:** John Fors, Natasha Strydom, William S. Fox, Ron J. Keizer, Radojka M. Savic

**Affiliations:** 1 Department of Bioengineering and Therapeutic Sciences, School of Pharmacy, University of California San Francisco, CA, United States of America; 2 InsightRX, San Francisco, CA, United States of America; University at Buffalo - The State University of New York, UNITED STATES

## Abstract

Standard treatment for active tuberculosis (TB) requires drug treatment with at least four drugs over six months. Shorter-duration therapy would mean less need for strict adherence, and reduced risk of bacterial resistance. A system pharmacology model of TB infection, and drug therapy was developed and used to simulate the outcome of different drug therapy scenarios. The model incorporated human immune response, granuloma lesions, multi-drug antimicrobial chemotherapy, and bacterial resistance. A dynamic population pharmacokinetic/pharmacodynamic (PK/PD) simulation model including rifampin, isoniazid, pyrazinamide, and ethambutol was developed and parameters aligned with previous experimental data. Population therapy outcomes for simulations were found to be generally consistent with summary results from previous clinical trials, for a range of drug dose and duration scenarios. An online tool developed from this model is released as open source software. The TB simulation tool could support analysis of new therapy options, novel drug types, and combinations, incorporating factors such as patient adherence behavior.

## Author summary

A comprehensive *in-silico* model of pulmonary tuberculosis successfully predicted previous clinical trials and could simulate future therapeutics.

## Introduction

Standard treatment for active pulmonary infection with *Mycobacterium tuberculosis* (TB) usually involves lengthy therapy based on four different drugs taken over 6 months. Adherence has been shown to be a major predictor of treatment failure and shorter-duration therapy has been suggested to lower risk of treatment failure and bacterial resistance [1–4]. Pharmacokinetic/pharmacodynamics (PK/PD) mathematical models for TB have mostly focused on short-term drug effects during the initial ten days of treatment rather than overall therapy outcome. Most earlier *in-vitro* experiments and *in*-*silico* simulations of TB drug effectiveness have been based on monodrug therapy, even though standard patient treatment for TB involves four or more drugs. Further, previous simulation models often have been relatively deterministic, and done at individual patient level, thus offering limited ability to assess therapy outcomes on a broader population basis [5–7].

Short- and long-term infection dynamics of TB are believed to be strongly influenced by immune system response of an affected patient. Goutelle et al., provided an integrated simulation of TB inflammation and immune response [8]. However, the study scope is limited to rifampin mono-therapy during the initial 2 to 14 days of drug therapy. In the real-world clinical setting, multi-drug therapy is needed to control risk of development of bacterial resistance. Additionally, the effectiveness of different antibacterial drugs is thought to have different level of bacteriostatic and bactericidal effect on fast- vs. slow-growing bacteria, and will also depend on bacteria population in distinct compartments, such as inside vs. outside of macrophages [9].

The primary objective of this study was to establish an integrated PK/PD population-based *in-silico* model, incorporating immune system and bacterial resistance dynamics, to estimate predicted relative effectiveness of drug therapy alternatives for pulmonary tuberculosis infection. This paper describes the resulting integrated simulation model, underlying mathematical formulation, parameter selection, numerical and computational methods, and validation and calibration with experimental data and several clinical drug trials.

### TB drug considerations for building the model

The unusual cell wall of the bacterium, and its ability to persist inside infected macrophages, renders many types of antibiotics effectively useless. Standard drug therapy for pulmonary tuberculosis infection in adults, with no suspicion of multi-drug resistance (MDR), involves four drugs. Daily rifampin 600 mg, isoniazid 300 mg, pyrazinamide 1500 mg and ethambutol 1200 mg for two months’ intensive therapy followed by a continuation phase of rifampin 600 mg, and isoniazid 300 mg, daily for 4 months. It is recommended these drugs be given as directly observed therapy (DOT), when possible [10].

Rifampin hinders bacterial DNA-related RNA synthesis by inhibiting bacterial DNA-dependent RNA polymerase by binding and physically preventing extension of RNA [11]. The precise intracellular concentration dynamics in macrophages is not fully understood, and may be affected by the local environment and metabolic state of macrophages and bacteria. Rifampin is believed to act on both intracellular and extracellular bacteria, and it is believed to have both bactericidal and sterilizing effect [12]. Rifampin will bind to human plasma proteins at around 85–90% [13]. Rifampin is an inducer of many enzymes of the cytochrome P450 superfamily—resulting in significant auto-induction leading to approximately twice steady state clearance [14,15].

Isoniazid is a prodrug, activated by catalase-peroxidase enzyme KatG in *M*. *tuberculosis* [16]. The drug inhibits synthesis of mycolic acid, needed to maintain the bacterial cell wall. Isoniazid is considered to be highly bactericidal on rapidly dividing extracellular mycobacteria, but only marginally bacteriostatic on intra-cellular bacteria, and minimally effective on slow-growing bacteria [17,18]. Metabolism of isoniazid is affected by an individual’s acetylation rate—*fast* vs. *slow* acetylators. Fast acetylators have approximately twice the clearance rate of slow acetylators [19].

Pyrazinamide is a prodrug inhibiting growth of *M*. *tuberculosis*, and can act both inside and outside of macrophages. The active form, pyrazinoic acid, is believed to accumulate inside bacteria, and reach a relatively higher concentration at acidic pH [20]. Pyrazinoic acid binds to the ribosomal protein S1 (RpsA) and inhibits trans-translation, and it is thought to inhibit synthesis of fatty acids [21,22]. It is considered mostly effective on slowly replicating “persisting” bacteria found in pulmonary lesions often considered to result in anaerobic conditions [20]. The activity on slow-growing bacteria is opposite that of many other drugs used in TB therapy, which act mainly on actively growing bacteria. Pyrazinamide’s accumulation in epithelial lining fluid has been observed as relatively high [23], while other studies looking at lesion and lung tissues showed significantly lower concentrations in lung tissue compared to plasma [24].

Ethambutol is believed to be mainly bacteriostatic [25]. The drug is believed to work by inhibition of arabinosyl transferase, interfering with synthesis of arabinogalactan, thereby disrupting formation of the bacterial cell wall [26]. Changes in the structure of the mycobacterial cell wall may increase permeability to other drugs.

### Immune response considerations

TB infection can lead to a host immune-bacteria reaction resulting in formation of nodules of inflamed tissue. Over time these nodules can expand and evolve to distinct granulomatous lesions. Lesions can become increasingly fibrous and even calcified–and this may help contain the bacteria, but also may provide a more protective microenvironment promoting sustained bacterial survival. Granuloma lesions are highly heterogeneous, depending on multiple factors such as size, vascularization, and caseation–affecting drug diffusion and concentration characteristics [27]. Recent analysis of dissected lung samples suggest drug diffusion and accumulation in granuloma may vary greatly among different commonly used TB drugs [28,29]. The precise mechanisms underlying the varied penetration of TB drugs are still being investigated [27].

The immune system response to infection with *M*. *tuberculosis* can be understood in terms of the interaction between bacteria, immune cells in alveoli of the lung, and lymph nodes. The primary mode of infection is per aerosolized bacteria entering the lung. Upon infection, resident macrophages undergo a sequence of transformation steps–from recruitment, infection, to activation. As an increasing number of macrophages are recruited, this triggers release of cytokines, including IL-4, which in turn regulate macrophage activation. The macrophages, not able to clear the infection, may become chronically infected. Chronic infection ultimately can result in killing of the macrophage which releases TB bacteria contained within it, which then can go on to trigger extended infection [30].

The University of Michigan group has extensive experience modeling within-host infection, summarized in their review [31]. Their models are able to reconstruct the immune response involved in forming granulomas and has advanced to an agent-based model that provides 2D and 3D spatial models of the lung parenchyma, immune cells and their interaction over time [32,33], and they have investigated various regimen optimization approaches within their computational model (*GranSim*) [34]. We considered one of their simpler models as a scaffold to add PK/PD interaction [30].

### Multi-drug considerations

Previous studies of commonly used TB drugs have shown kill rates decline over time [35]. This decline may occur due to multiple factors, such as growth of resistant sub-populations of bacteria and overall reduction in absolute remaining number of bacteria. The decline in kill rate is more pronounced with mono-drug therapy, likely due to its inherent higher risk of bacterial resistance. Multi-drug therapy is a key aspect of effective long-term TB treatment since mono-drug therapy has been show to lead to increased bacterial resistance, sometimes in as short a period as a few weeks or months [20]. One inherent complication of analyzing the effectiveness of multi-drug therapy is the difficulty in determining specific contributions of individual drugs. Furthermore, drug combinations may result in both synergistic and antagonistic effects at different dose levels. TB drug combinations may be slightly antagonistic at lower dose levels and neutral or slightly synergistic at higher dose levels as some studies suggest [36].

## Results

### General

The simulation system was based on a series of connected underlying models, each representing distinct portions of the overall dynamic host-pathogen-drug system. Major sub models included:

1. Bacterial infection and growth
2. Immune system response
3. Patient drug therapy adherence
4. Pharmacokinetics, including drug diffusion into macrophages and granuloma lesions
5. Pharmacodynamics
6. Bacterial drug resistance
7. Population modeling and patient outcomes

**Fig 1:**
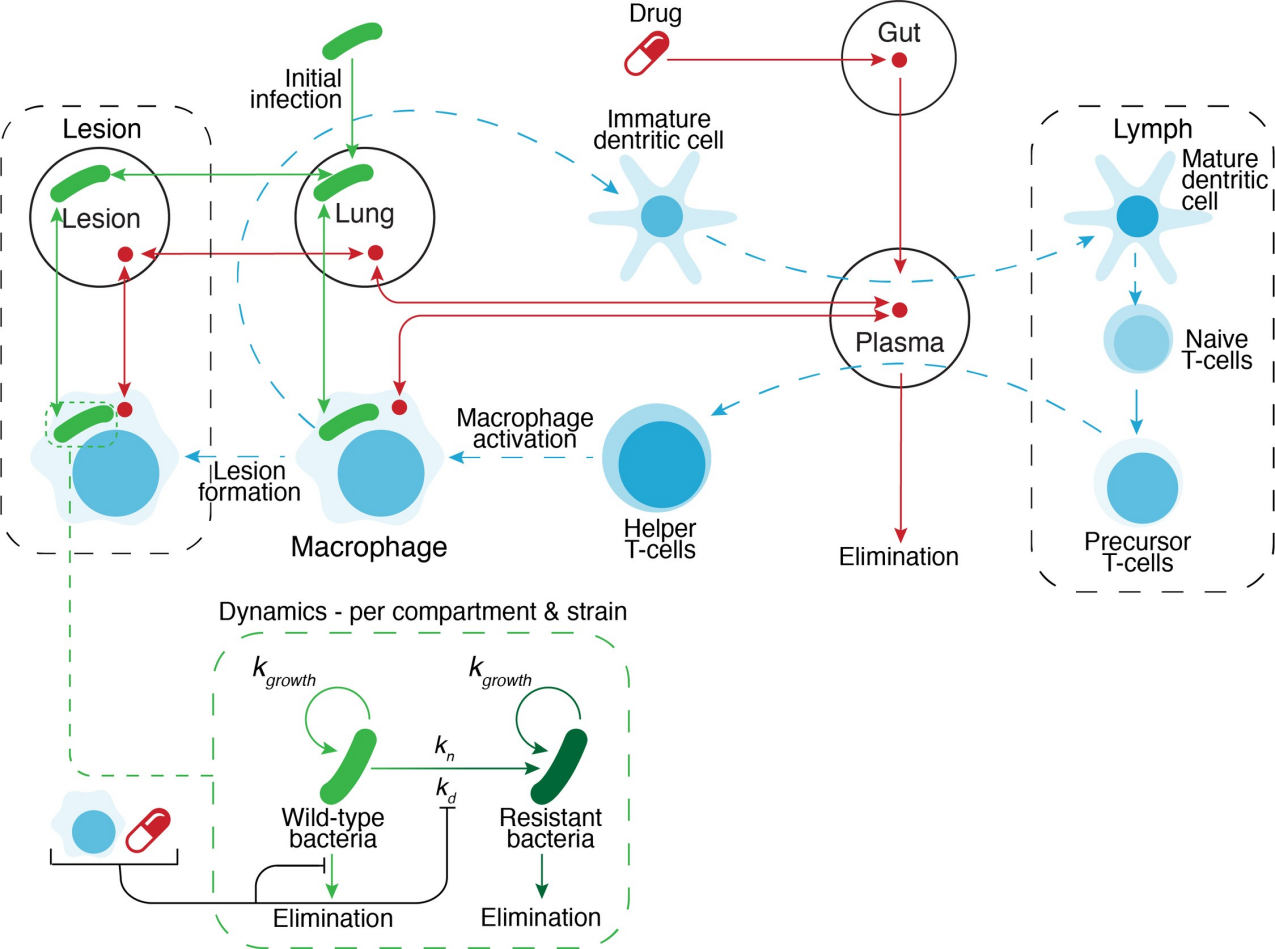
Complete structural model. Bacterial dynamics is illustrated in the Bacterial dynamics cutout and is modelled in each respective compartment. The immune system response is based on a model from literature[30] and is illustrated in blue. The immune system follows activation of macrophages by the initial infection, leading to a cascade of additional white cells in the lymph, finally leading to increased macrophage involvement and the formation of lesions. Drug therapy is illustrated in red and can be modelled with multiple drugs and their respective penetration into lesion, macrophages and lung. Drug resistant bacteria (dark green) is modelled using both natural and drug pressure mutation rates (*k*_*n*_ and *k*_*d*_ respectively). Bacteria killing is determined by both immune and drug related increase in bacterial elimination.

The host system was defined as a series of linked compartments, including plasma, lung, lymph node, extracellular vs. intracellular macrophages, and inside/outside of granuloma lesions. The simplified structural model is illustrated in Fig 1. The different sub models apply to one or several of these compartments. The immune model [30] and description of bacterial infection and growth is briefly described in the Materials and Methods section and the equations used for the immune system is presented in the S1 Text document. The other subsections are described in detail with equations in their specific subsections of Materials and Methods. The section on pharmacokinetics provides a more detailed description of drug transfer between compartments, and use of effect compartments to model drug concentration inside macrophages and lesions.

Much of the ultimate utility of an *in-silico* simulation model generally hinges on its ability to consistently generate patient outcomes similar to real-world clinical results. The primary response metric for the simulation model was the share of the patient population without TB infection (cleared TB) defined by the final number of bacteria present at the end of the drug therapy period. The criteria of the response metric are defined in Table 1 and specifically take into account bacteria in granulomas with differentiation between fully cleared (<1) and latent TB (between 1 and 1000). The model is able to differentiate between an active infection and latent TB which is important when considering that patients may reach a point of low enough bacterial burden to suppress the infection but are still at risk of relapsing at a later time. Clinical trials show that standard 6-month multi-drug TB therapy can remove TB infection in approximately 95 percent of affected patients in a population[1]. Validation of outputs from the simulation model is described in four steps, 1) check basic simulation model outputs, 2) review simulation of standard TB therapy, 3) comparison with a wider set of clinical trials, and 4) test impact of other drug therapy parameters and population characteristics.

**Table 1:** Rules for patient drug therapy outcomes.

Infection Status	Extracellular Granuloma (CFU/mL)	Intracellular Granuloma (CFU/mL)	Patient Drug Therapy Outcome
Not infected	0	0	N/A
TB infection	> 1 or > 1000	> 1 or > 1000	Failure
Latent TB	< 1	1 < x < 1000	Success
Cleared TB	< 1	< 1	Success

### Basic simulation outputs

**Fig 2:**
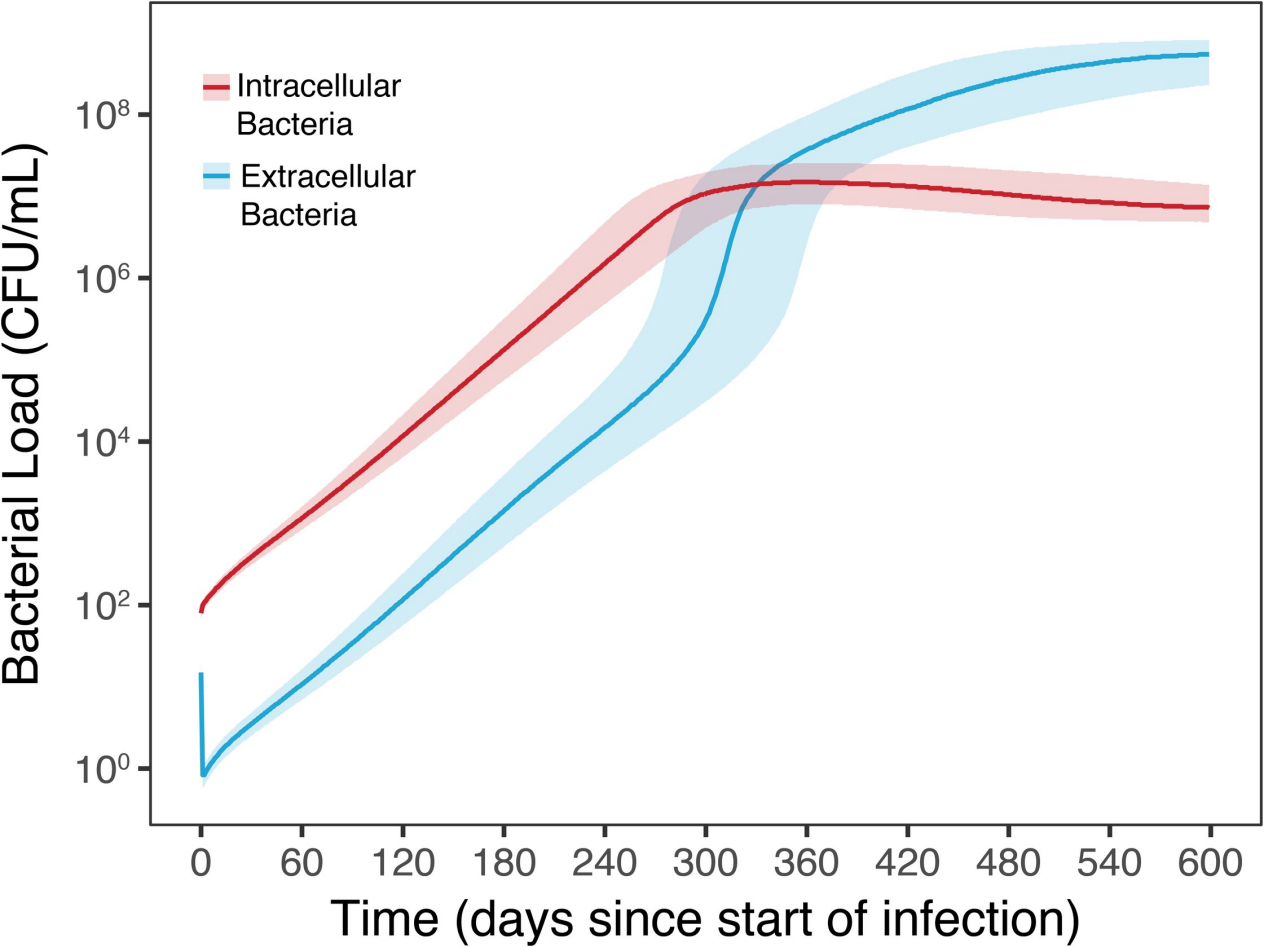
Bacterial dynamics of simulated TB infection. Intra- (red) and extracellular (blue) bacteria count in lung tissue for TB infection without drug therapy, for 5000 patients, with infection starting on day 0. Respective shaded areas represent first and third quantiles of population.

Simulation of bacterial dynamics for TB infection without drug therapy is illustrated in Fig 2. After a bacterial inoculate of 100 CFU/mL infects extracellularly, rapid intra- and extracellular bacterial growth is triggered. The infection approaches a steady-state level after 9 (270 days) to 12 months (360 days). Inoculation size was within the suggested range from Marino et al., 2004. Untreated infection results in steadily growing bacterial population, both intra- and extracellularly, reaching steady state level after 9 to 12 months, in line with the validated model from Marino et al., 2004 [30]. The simulation model generates unique pharmacokinetic profiles, per drug and patient, as shown in S1 Fig. Pharmacokinetic profiles summarized at a population level for all active drugs are shown in S2 Fig.

### Simulation of standard TB therapy

**Fig 3:**
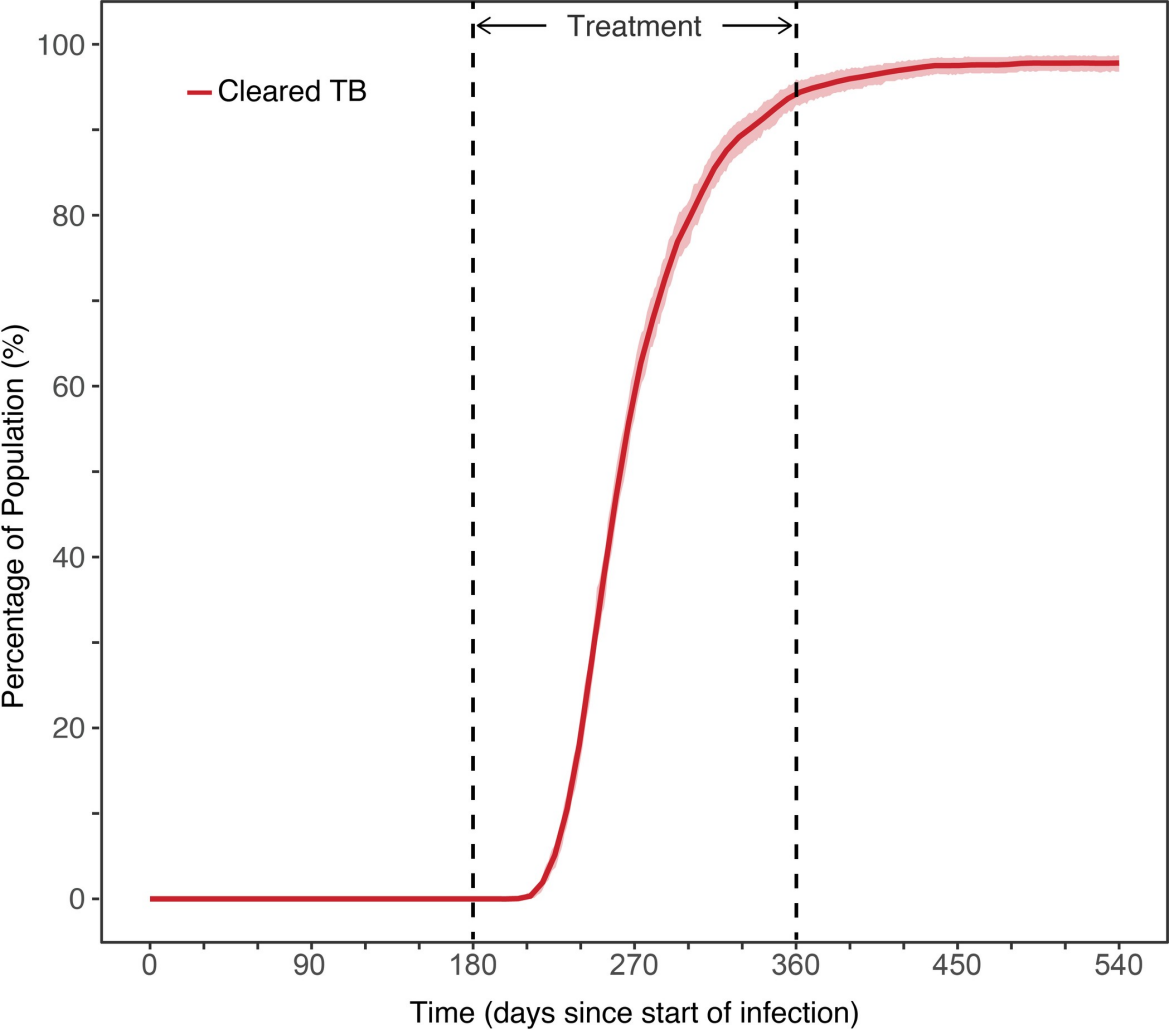
Population TB treatment outcome with standard drug therapy. Summary of outcome of median standard drug treatment of TB infection, based on simulation of 5000 patients, with standard drug therapy (2HREZ/4HR). The TB infection occurs on day 0, and drug therapy is started on day 180.

**Fig 4:**
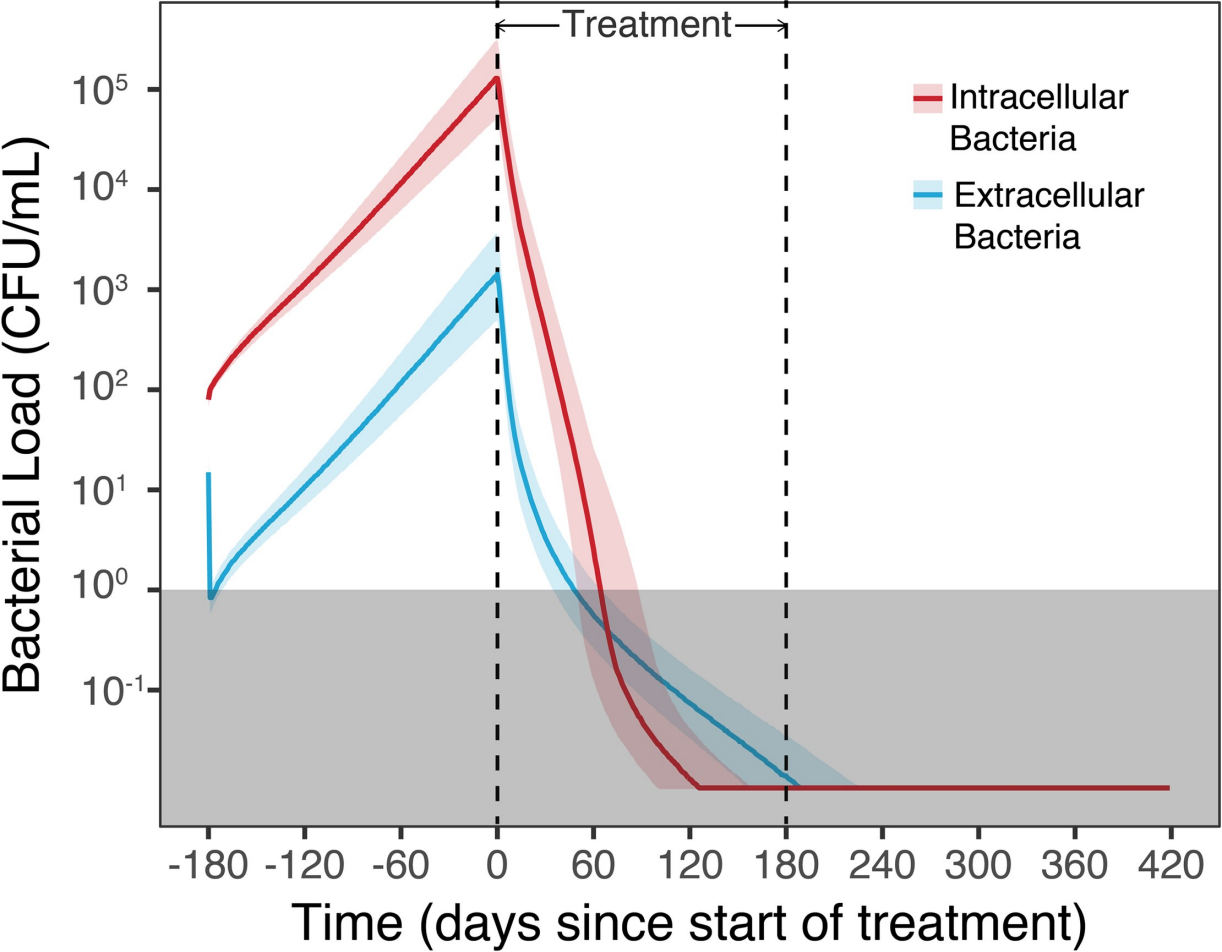
Intra- vs. extracellular bacterial load in TB infection. Intra- and extracellular bacteria count in lung tissue for TB infection with standard drug therapy (2HREZ/4HR). The TB infection occurs on day -180, and drug therapy is started on day 0, triggering rapid intra- and extracellular bacterial death. The grey shaded block represents the cut-off of CFU/ml equal to 1 for a patient to be considered cleared of bacteria at the end of treatment.

Simulation of outcome of standard drug therapy (2HREZ/4HR) with perfect patient adherence, suggest 70% of patients are cleared of infection after approximately 60 days, and this grows to most patients (~95%) after 120 to 150 days, with a small increase in cleared infection after treatment, due to influence from the immune system as shown in Fig 3. This outcome level is generally consistent with results from major clinical trials, such as the RIFAQUIN study [4]. Intracellular bacterial load is higher compared with extracellular bacteria, and requires longer time to clear, as illustrated in Fig 4. It is possible bacteria inside infected macrophages form a continued reservoir that may sustain extended infection.

### Comparing TB simulation results with previous clinical trials

Simulation results for standard TB therapy were compared with several previous clinical trials that included variations of standard therapy, such as shorter or longer duration, increased or reduced dose, and daily vs. intermittent therapy. The analysis was an extension on a previous extensive review of TB clinical trials [37,38]. It is inherently challenging to compare absolute outcome levels for simulations compared with clinical trials, for trials performed 10 to 20 or more years ago. Therefore, the comparison was done based on change in outcome in alternative trial arms vs. standard TB therapy. Studies included in the summary analysis are provided in Table B in S2 Text.

The simulated therapy outcome was found to be mostly consistent with risk of relapse found in the summary of previous clinical trials [37], including

1. Standard 6-month TB therapy
2. Shorter 4-month TB therapy
3. Longer 9-month TB therapy
4. Intermittent TB therapy (with dosing 2/week during continuation phase).

**Fig 5:**
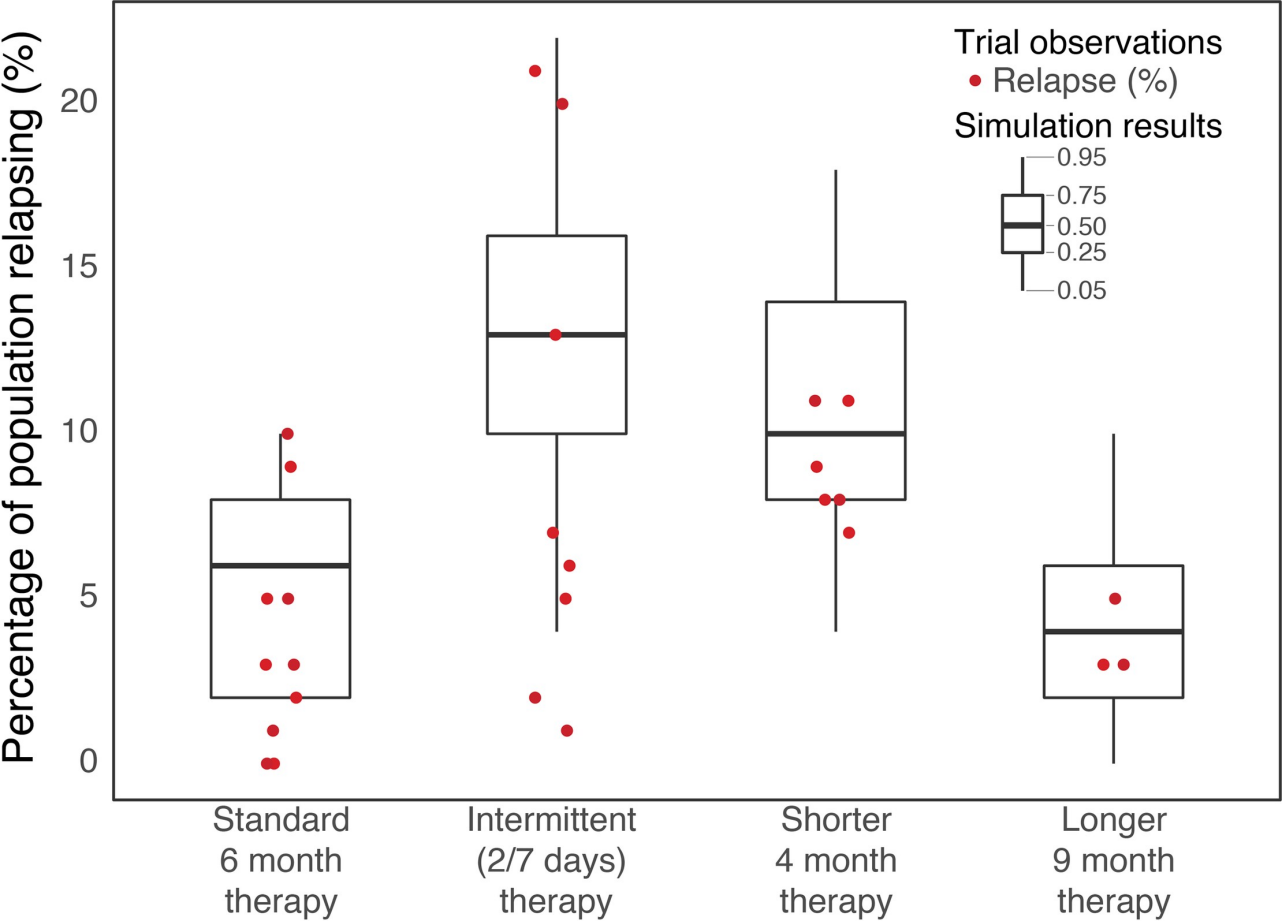
Comparison of therapy outcome for simulation vs. summary of previous clinical trials. Comparison of simulated population consisting of 5000 patients (boxplot) therapy outcome vs. summary of previous clinical trials in terms of risk of relapse as percentage of overall population grouped by changes in duration and frequency of standard therapy. Clinical trial data with individual therapy simulations used for this figure is summarised in the Addendum B.

In Fig 5 is shown the results of 5000 simulated patients in comparison to clinical trials, where clinical trial outcomes for 4-month, 6-month, and 9-month, and for intermittent therapy alternatives are based on differential relapse rates from 27 different regimens, shown in S2 Text. Intermittent therapies had daily dosing during intensive phase, and twice-a-week dosing during continuation phase.

### Simulating drug therapies and patient characteristics

The inherent flexibility and speed of an *in-silico* simulation approach allows for rapid evaluation of numerous different treatment parameters, such as drug combinations, drug dosing, therapy start time and duration, and patient adherence. Infection dynamics and patient outcomes for drug therapy variations are illustrated through a number of simulation scenarios.

### Impact of dose changes and therapy duration for drugs in standard of care regimen

**Fig 6:**
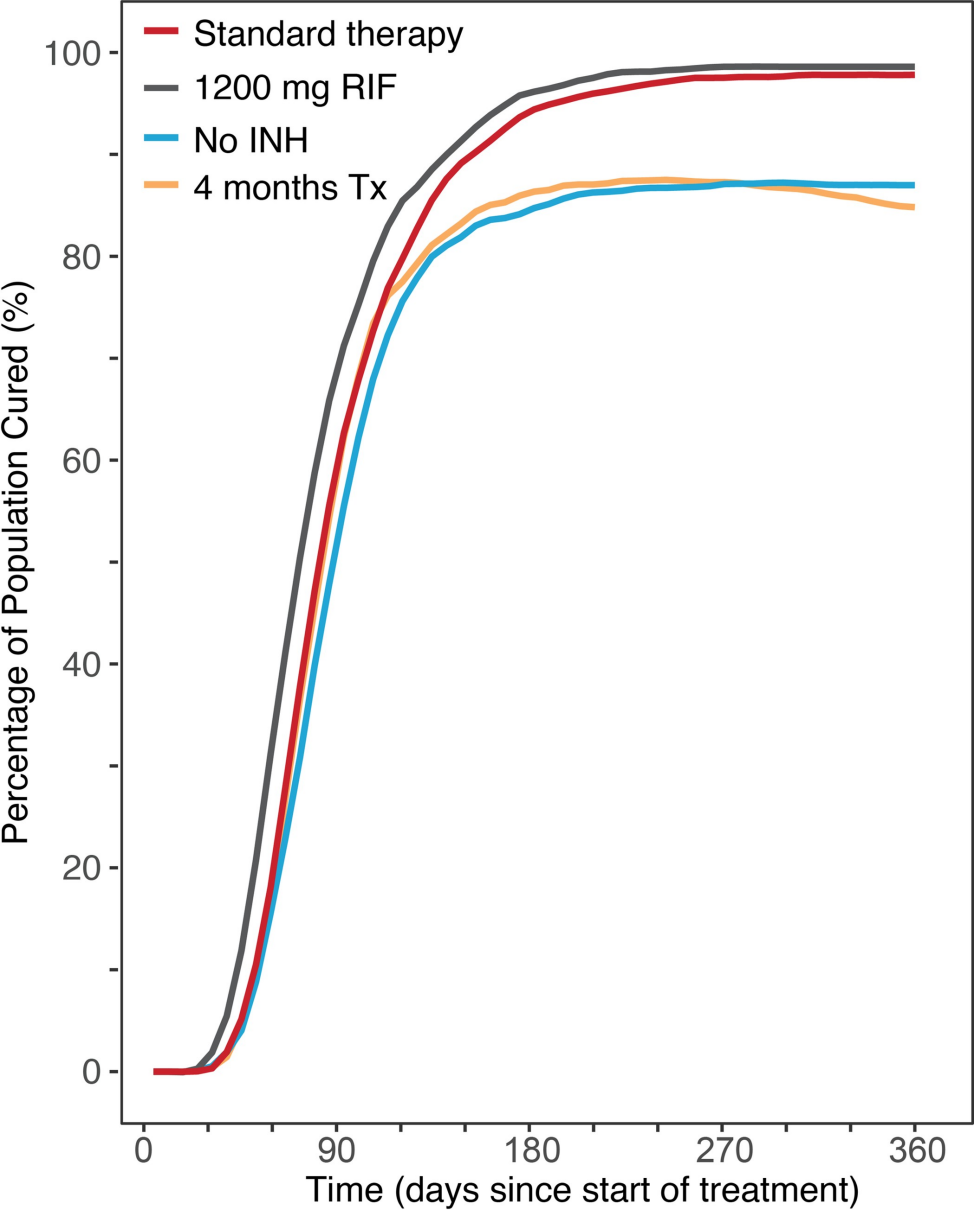
Reduced successful therapy outcome with variations of standard drug therapy. Comparison of patient outcomes for treatment of the median outcome of TB infection, with standard drug therapy (2HREZ/4HR) vs. median outcome of therapy excluding isoniazid, 1200 mg rifampin and reduced treatment time. Based on simulation of 5000 patients.

We evaluated the change in percentage of population cured when changes are made to standard therapy, including increased or decreased dose levels and therapy duration. Eliminating isoniazid from the regimen resulted in 87% of patients being cleared of disease. (Fig 6). Increased drug dose may drive more effective bacterial killing, but could also increase risk of adverse drug effects. It has been argued many patients may be able to tolerate increased dosing of rifampin, such as using a dose of 1200 mg rather than regular 600 mg, Fig 6. Simulations suggest increased dosing has potential to improve patient outcomes, but not allow for drastically shorter-duration therapy. A scenario with shorter overall therapy duration includes 2 months of intensive ethambutol, isoniazid, pyrazinamide and rifampin, followed by 2 months continuation phase of isoniazid and rifampin–using standard drug dose and schedule, results in approximately 12 percent reduced clearing of infection compared to 6 month treatment.

### Improvement in therapy start

**Fig 7:**
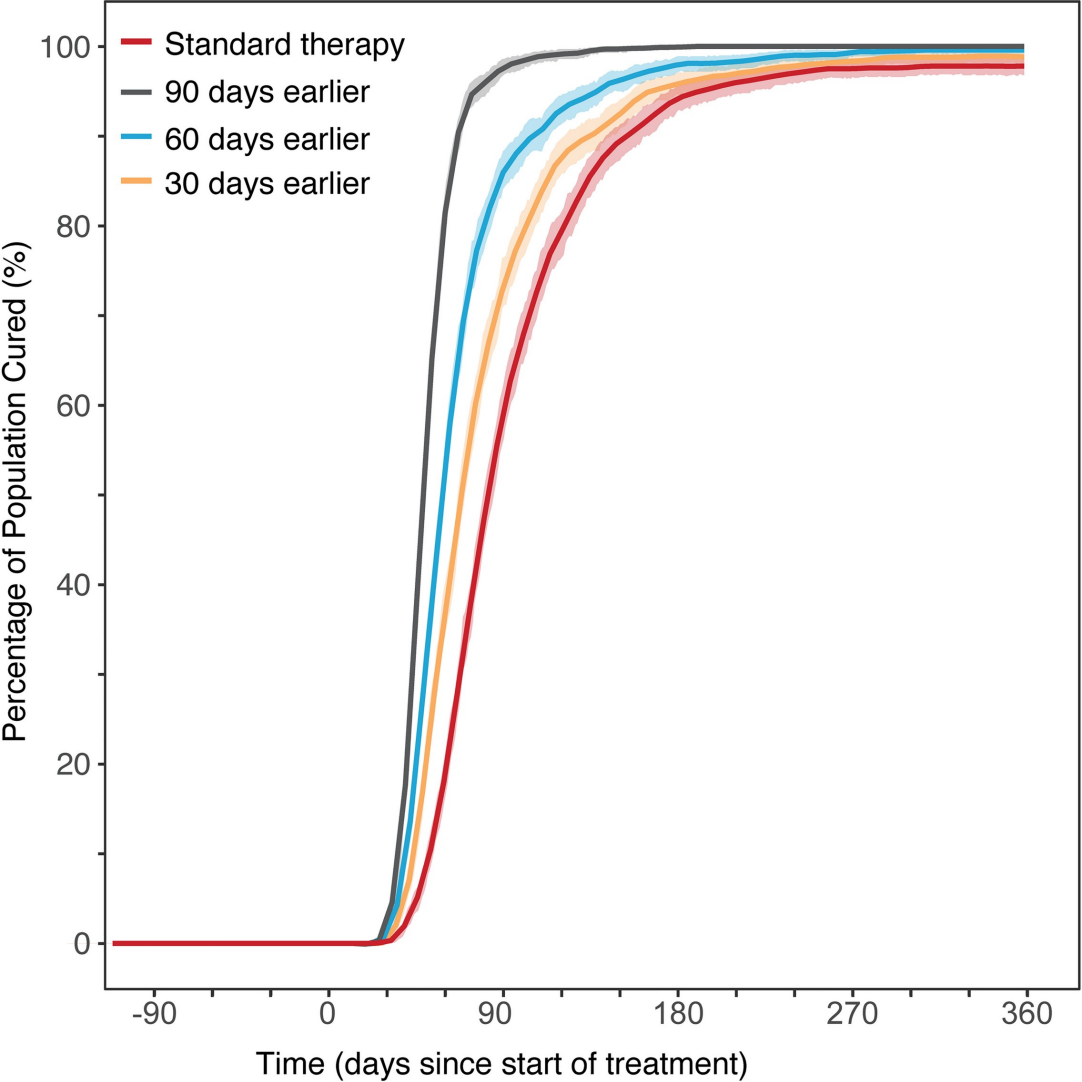
Population TB treatment outcome for different treatment start scenarios. Comparison of median patient outcomes for treatment of TB infection, with standard drug therapy (2HREZ/4HR) of earlier diagnosis and the impact on patient outcomes if treatment is started earlier. Based on simulation of 5000 patients. Shaded areas show the 95 percentile range of the simulations.

The time delay from initial infection to start of drug therapy, and overall duration of drug therapy can affect patient outcome. Simulations suggest that a shorter time from initial infection to start of drug therapy could decrease treatment duration. To clear 90 percent of infection for the default simulation of 180 days infection, 150 days are necessary. If treatment starts 30 days earlier then a 90% clearance of infection occurs 20 to 25 days earlier. Simulations for 60 and 90 days earlier treatment (i.e. after 120 and 90 infection days) also show improved slope with 90% patients treated in 100 and 60 days, Fig 7. All simulations used inter-individual variability on patient pharmacokinetics and disease progression as defined by their respective literature values.

### Effect of patient adherence

**Fig 8:**
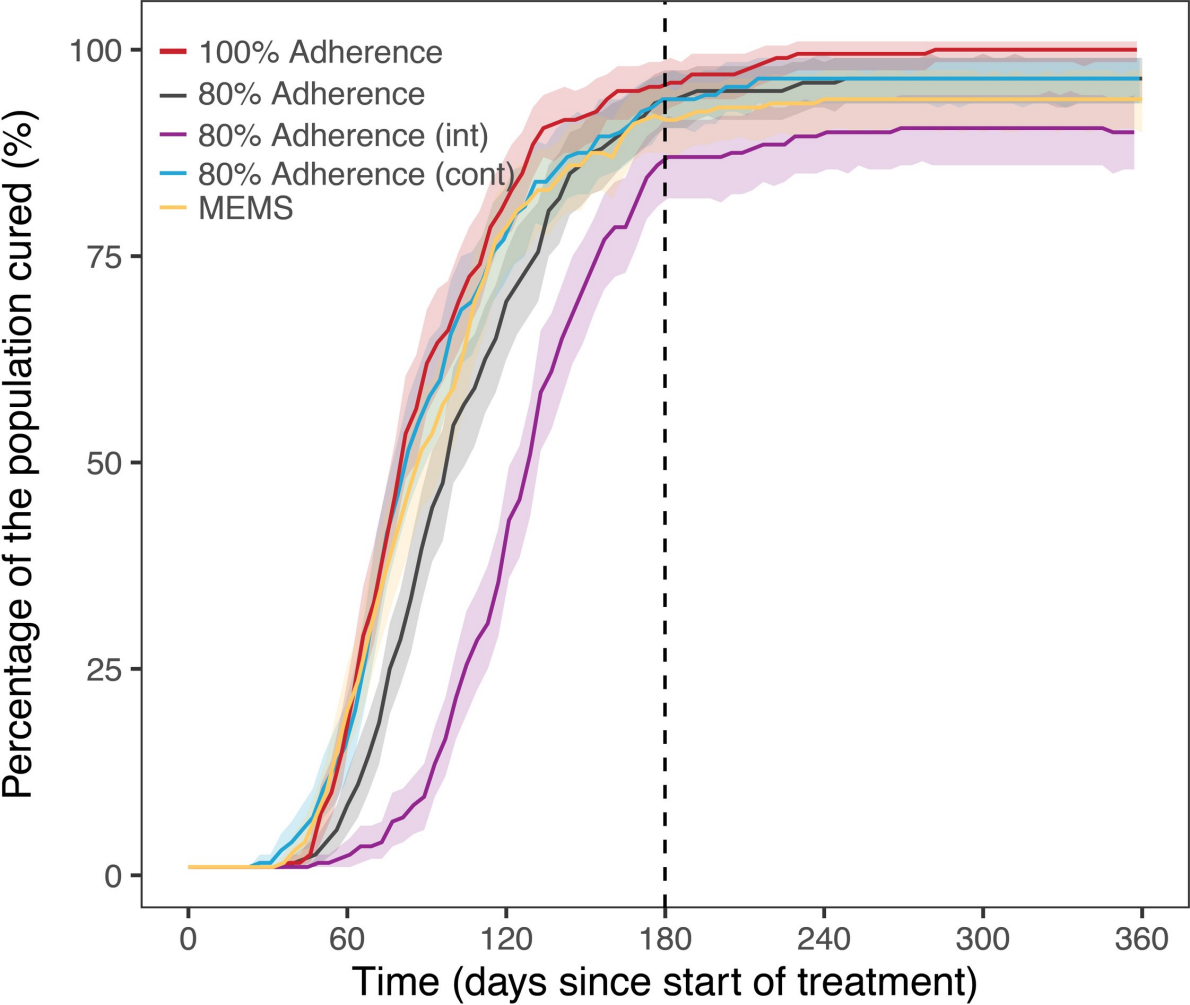
Population TB treatment outcome for different adherence patterns. Comparison of median patient outcomes for treatment of TB infection, with standard drug therapy (2HREZ/4HR) at 100% adherence, 80% adherence randomly spaced throughout treatment, 80% during intensive or continuation phase and simulations from medication event monitoring system (MEMS) data. Based on simulation of 5000 patients. Infection occurs 180 days before treatment.

Patient behaviors of adherence affect therapy outcomes [1]. We simulated 4 scenarios to evaluate the effects of missing doses during treatment. Missing 80% of doses (a cut off described by recent clinical trials as still adherent [2,3] randomly throughout the full course of treatment results in 5% less people cured. When the doses are missed more during the intensive or continuation phase of treatment, we observe much worse outcomes for those that missed doses during their intensive phase. We also evaluated data from real medication event monitoring system (MEMS) and found this data to share similar trends to our 80% doses missed in the continuation phase simulations, Fig 8.

### Effect of lowered immunity

**Fig 9:**
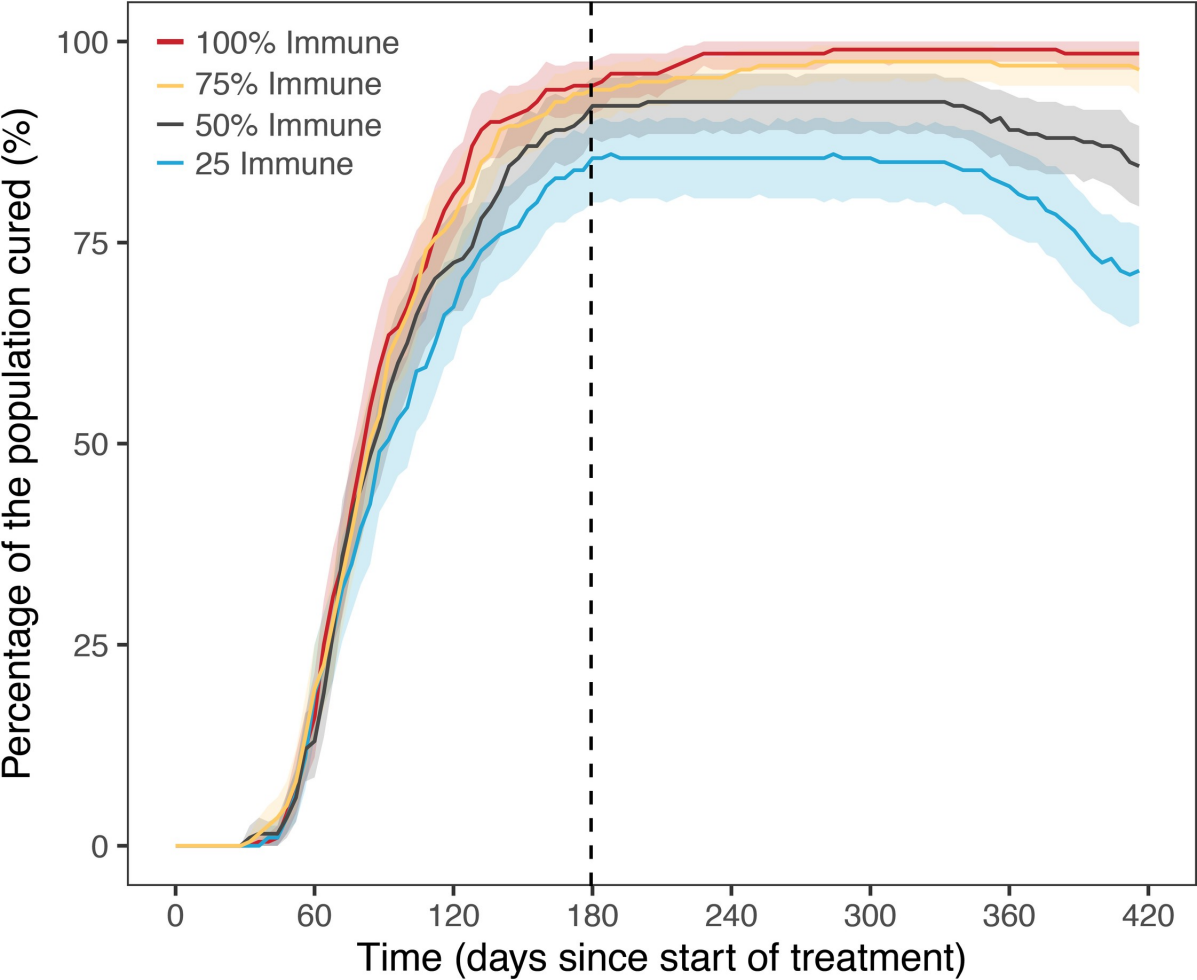
Population TB treatment outcome for reduced immune strength. Comparison of median patient outcomes for treatment of TB infection, with standard drug therapy (2HREZ/4HR) and patients with reduced immune response calculated as a percentage of full immune system present. Based on simulation of 5000 patients. Infection occurs 180 days before treatment.

It is common for TB patients to have comorbidities reducing overall immune system function, including but not limited to HIV infection. We simulated lower percentages of immune kill on bacterial dynamics to observe the magnitude of immune killing on our final outcomes. Simulations results suggest such patients may respond well to initial drug treatment for TB with seemingly only 10% reduced cure rate for those with severely reduced immune function. However, 6 months after treatment, the lack of immune system has a reduced ability to suppress long-term tuberculosis regrowth. In Fig 9 is shown how the number of successful outcomes decrease long term depending on the strength of the immune system.

### Role of monotherapy on resistance

**Fig 10:**
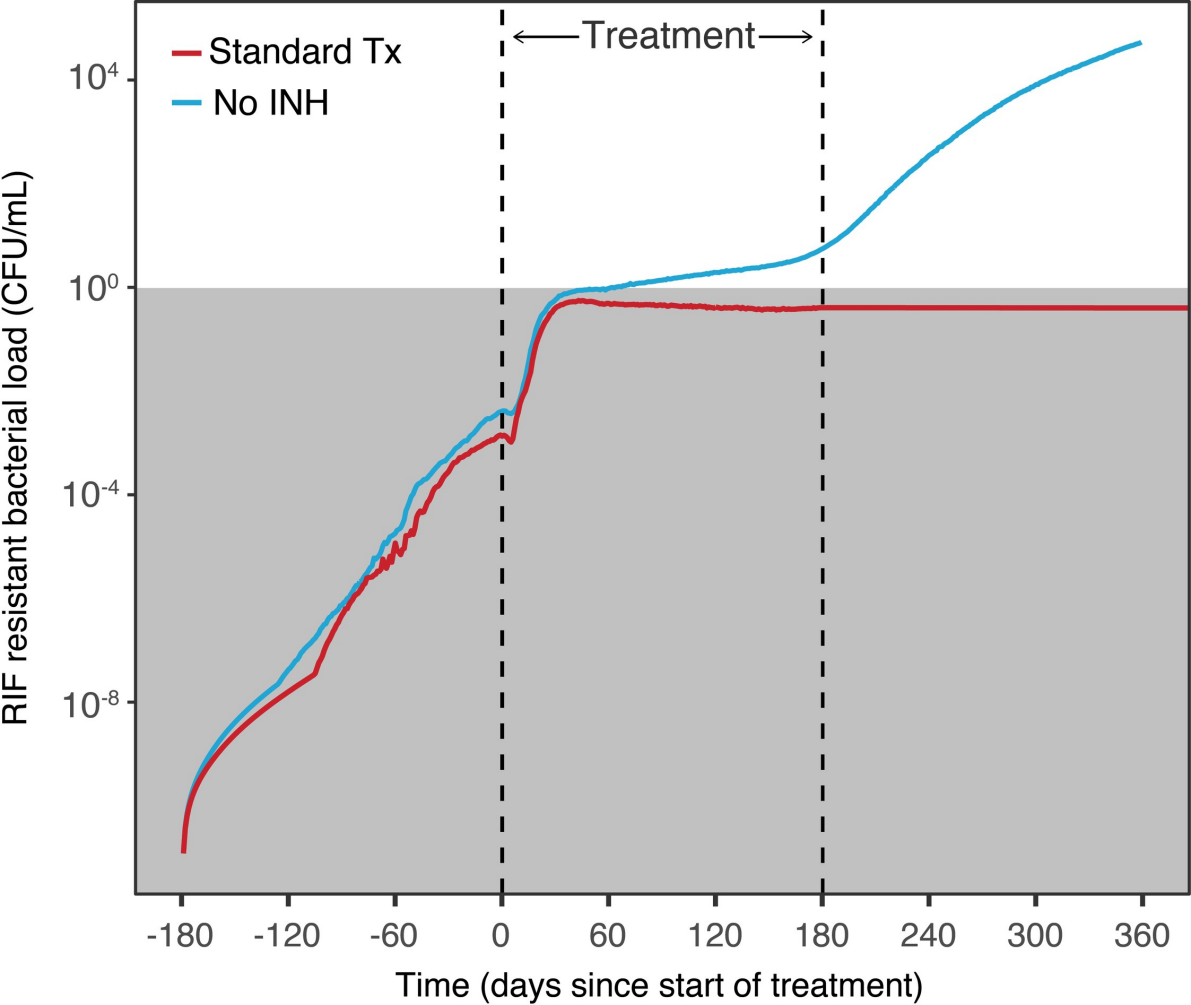
Faster growth of drug-resistant bacterial strains due to incomplete drug therapy. Comparison of growth of rifampin mono-resistant bacterial strain during treatment of TB infection, with standard drug therapy (2HREZ/4HR) vs. therapy excluding isoniazid. Based on simulation of 5000 patients. Infection occurs on day -180, and drug therapy is started on day 0.

We evaluated the change in bacterial resistance when removing isoniazid from standard therapy. As shown in Fig 8, treatment outcome is worse when no INH is present. Mono-drug therapy for TB is known to increase risk of bacterial drug resistance. Comparing rifampin resistant bacteria under normal standard therapy and without isoniazid on board, we see isoniazid acts to protect against resistant bacteria crossing above the threshold of our cleared definition. In the isoniazid absent scenario, rifampin resistant bacteria grows faster and is able to proliferate above the definition of cleared after treatment stops, continuing to grow and produce full blown resistance, Fig 10.

**Fig 11:**
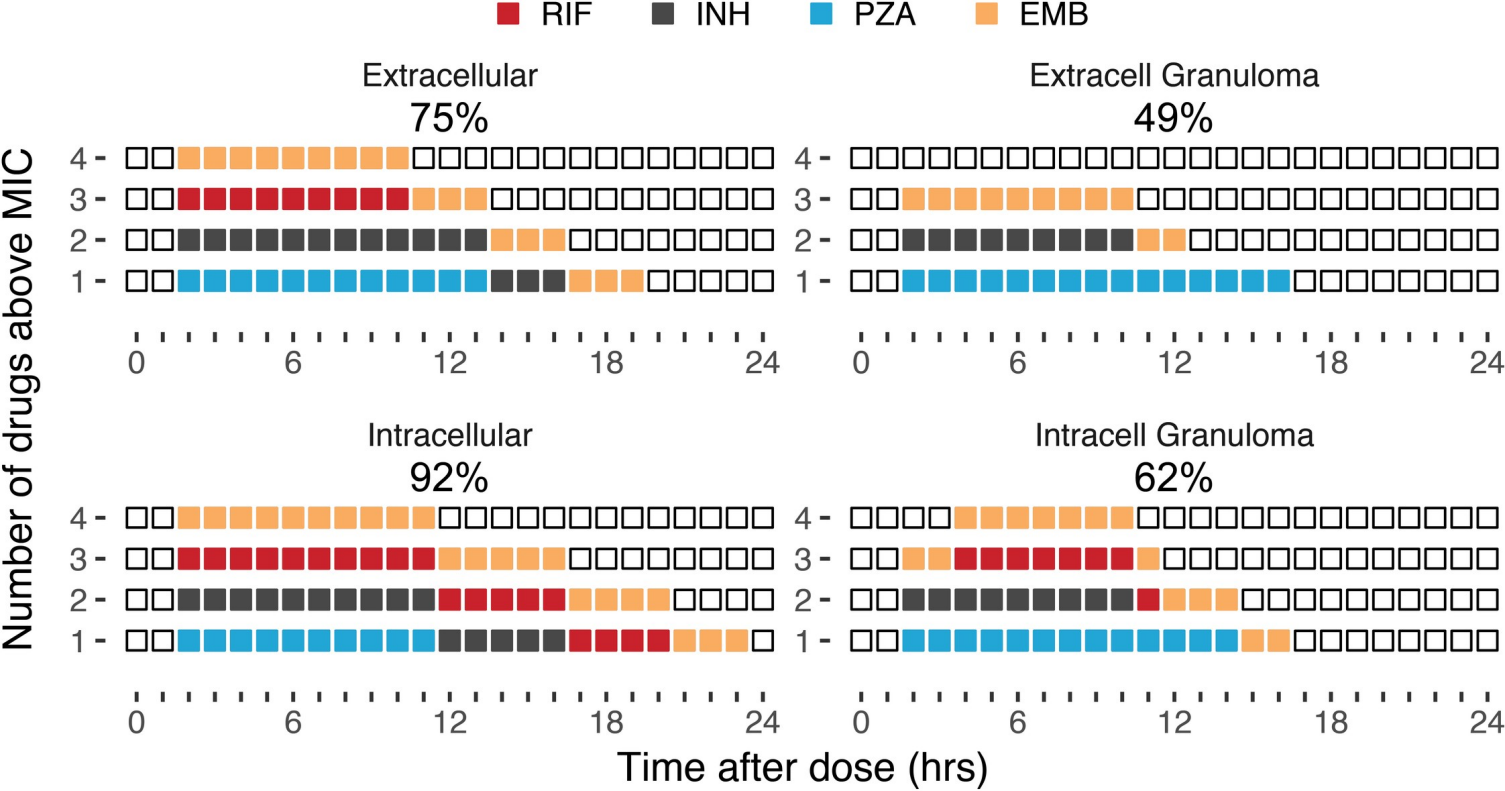
Number of drugs above MIC by compartment. Number of drugs above MIC at steady state of standard therapy drugs at steady state over a 24h dosing interval for 5000 simulated patients. Each colored block represents one hour that a drug is above its MIC. The drugs are stacked for each hour with different colors representing different drugs. Empty squares show no drug on board. The percentage of drug onboard hours vs expected drug hours is shown above each plot to compare total compartment exposure in a 24h period.

Considering all tuberculosis drugs might not have optimized pharmacokinetics and could result in long windows of monotherapy, we examined drug concentrations relative to their minimum inhibition concentrations (MIC) and see that drug coverage in granulomas are estimated between 49% and 62% of expected number of hours above MIC, Fig 11.

### Sensitivity analysis of parameters

**Fig 12:**
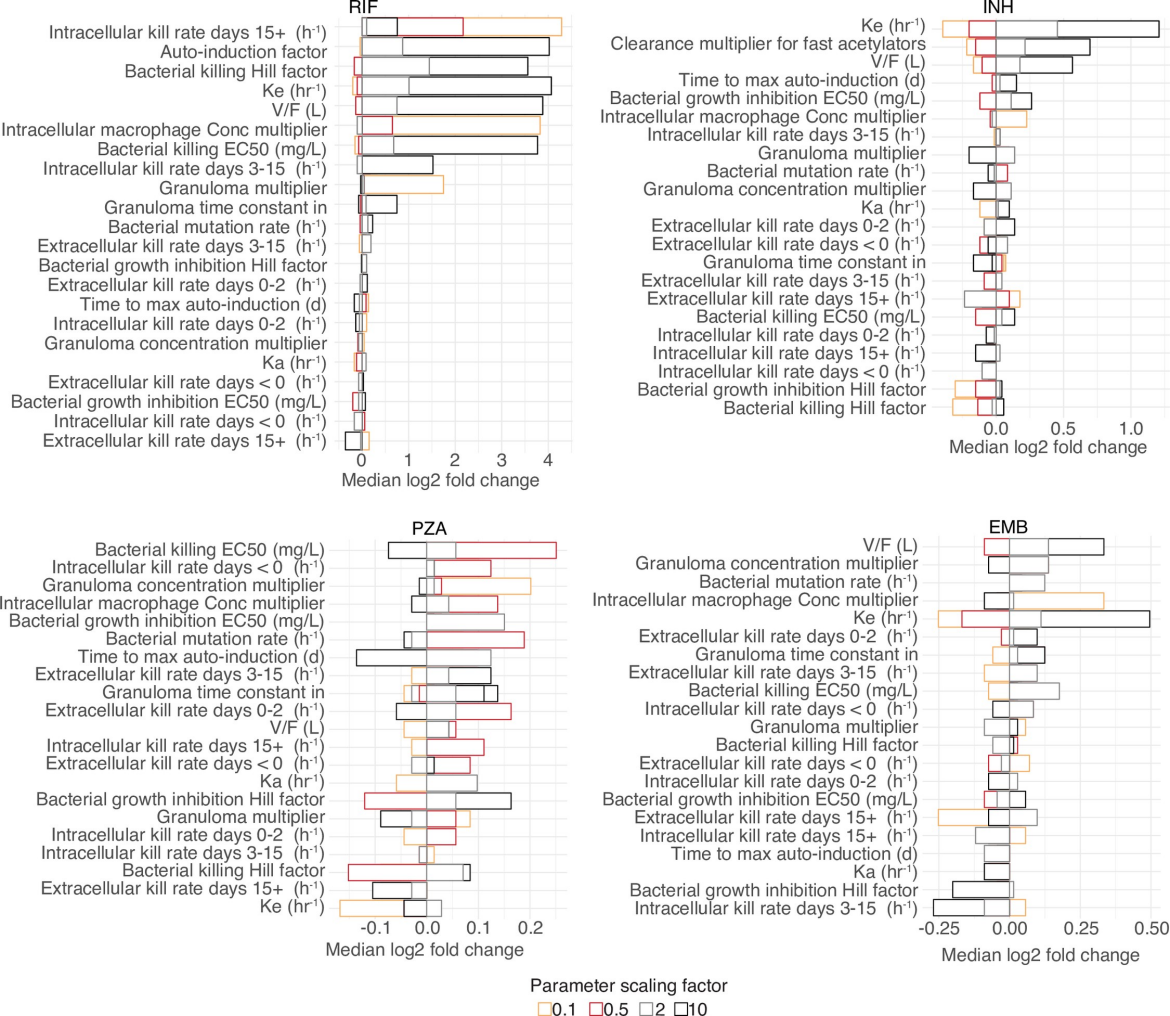
Sensitivity analysis of drug parameters. Parameters were scaled by factor 0.1, 0.5, 2 and 10, and the final change in percentage of the population with TB (mean 5% under normal conditions) recorded to measure individual parameter impact on TB outcome.

A sensitivity analysis was performed to measure the impact of individual parameters on the number of unfavorable outcomes expressed as the percent change in patients relapsing, including drug parameters Fig 12, and initialization and immune parameters in supporting S3 Fig. For the drug parameters, rifampin parameters had the largest impact in increasing unfavorable outcomes at the end of treatment. The parameter defining intracellular kill rate after 15 days had a greater than 16-fold change in unfavorable outcomes when scaled by 0.1 and changing the bacterial killing hill factor saw an increase in unfavorable outcomes by approximately 11-fold. This is an important consideration as estimating this parameter in the hollow fiber model occasionally results in high standard deviation and could lead to significant changes in the outcomes. Potentially restructuring to a binding kinetics model could improve precision in our model [39]. An increase in parameters related to clearance, (elimination rate and auto-induction factor) resulted in a 16-fold increase in unfavorable outcomes. Isoniazid parameters did not have as large an impact as rifampin and the highest observed change was when isoniazid elimination was increased resulting in a greater than 2.5-fold increase in the total population left with unfavorable outcomes. Pyrazinamide and ethambutol showed little to no change in outcome, with the largest change observed for EC_50_ at a 1.2-fold increase in unfavorable outcome.

The initialization parameters showed that adherence was the largest contributing parameter to the number of patients with unfavorable outcomes at the end of treatment. Reducing the adherence to 0.1 resulted in zero patients cured. An adherence of 0.5 resulted in 18.76-fold less patients cured. Other important parameters contributing to a large increase in unfavorable outcome was an increase in bacterial kill rate, drug killing level inside granuloma vs outside of granuloma (granuloma kill level) and the threshold of active infections (free bacteria level).

The immune parameters showed that the immune system had a large impact on total unfavorable outcomes at the end of treatment with 7 out of 17 parameters related to macrophage response showing a 11-fold or greater decrease in unfavorable outcome. The 2 highest impact parameters were related to macrophage infection. Cytokines showed an increase in unfavorable outcomes of greater than 8-fold for 3 out of 20 parameters, and variables affecting IL-12 production having the highest impact. The parameter responsible for the elimination of T-cells was responsible for the highest increase in unfavorable outcomes of all the immune parameters at 16.5-fold. Parameters related to differentiation and migration of T-cells had an increase in unfavorable outcomes greater than 8-fold. Parameters responsible for draining lymph nodes and dendritic cells had less impact than other immune parameters and the greatest increase in unfavorable outcome was seen for the elimination of mature dendritic cells (MDC) with an approximately 6-fold increase. Parameters controlling bacteria in the model responsible for the highest change in unfavorable outcome were related to bacterial growth and the number of bacteria released from macrophages after apoptosis with a greater than 16-fold change.

### Web application and R package

**Fig 13:**
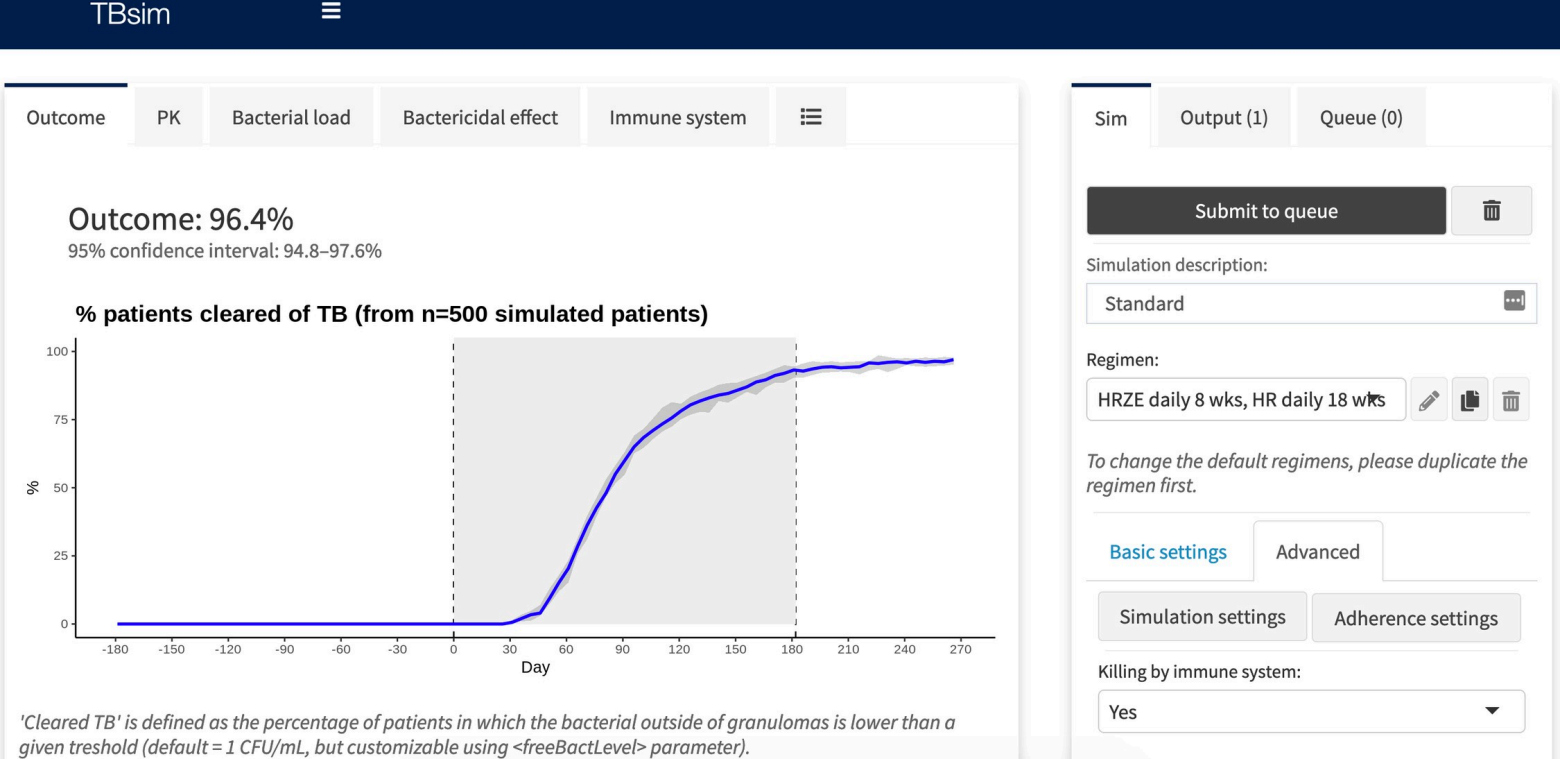
Screenshot of web application. Graphical user interface of the TBsim tool. User can adjust regimen, drug parameters, adherence, and immune and resistance inputs. Outputs include pharmacokinetic profiles, bacterial load in intra and extracellular compartments, and immune system plots.

A web application was developed to provide a graphical interface of the model, Fig 13, available at www.saviclab.org/systems-tb/. The application code and the underlying R package allows simulation of individual patients as well as populations of patients. All settings related to the considerations mentioned in the sections above can be customized in the web app, or in R using functions exposed in the package. New drugs and drug regimens can be created and used in simulations, and various adherence patterns can be selected and customized.

## Discussion

This study presented an integrated systems pharmacology model and simulation tool to support prediction of outcomes of different therapies for pulmonary tuberculosis infection. The simulation model allows rapid and flexible assessment of impact of various drug therapy and population parameters, such as drug selection, drug dose and dosing patterns, multi-dose combinations, and therapy start time and duration.

### Simulation results vs. real-world outcomes

Tuberculosis is an unusual pathogen given its relatively slow growth rate, its ability to survive and multiply inside macrophages, and interaction with the host’s immune system leading to granuloma lesions. The simulation was based on a multi-compartment model with various immune cell types, cytokines, and TB bacteria strains. The simulated time estimate needed to resolve TB infection was consistent with therapy timelines seen in clinical trials. Further, the simulated population therapy outcome was similar to clinical trial results, i.e., approximately 95% resolution among infected individuals receiving standard 6-month TB therapy. There are numerous ways the simulation model may be further extended to allow for even closer alignment with real-world patient outcomes, such as pharmacokinetic model considerations, bacterial resistance considerations, and bacterial fitness over time.

### Optimizing standard therapy

Our simulations on standard therapy showed that the current standard therapy is well optimized. Number of patients cured might not benefit from dose changes as the increase in population cured was slight when compared to standard therapy. Decreasing therapy duration results in a loss of 15% patients cured and increased therapy is able to kill all. This aligns with recent work that some patients may benefit from longer duration of treatment and others could be cured after 4 months, based on disease severity. The tool could further help regimen development by better optimizing intensive and continuation phase drug selection and durations of these phases. Toxicity would have to be carefully considered however and this is not taken to account in the model. Future optimization of the tool could include toxicity thresholds to provide safer and effective regimens by allowing more exploration of the therapeutic margins of these drugs.

While we found the quantitative systems pharmacology methodology appropriate for our objectives, alternative modeling methods investigating drug regimens have shown new ways to explore multi-drug therapies [40]. A model-based adaptive optimal design could navigate the risk-benefit space and provide optimized regimens more accurately than adding toxicity thresholds to our existing model.

Our model does not stratify patients based on their disease severity but does allow for variance of disease development. In future it could be interesting to see patients stratified by their risk groups and cater regimen selection to at risk groups, rather than a one-size-fits all approach.

The best change in outcomes was seen with earlier start of treatment. This suggests that though dose and duration changes of standard therapy might be slight, we can hope to see real therapy improvements with improved biomarker detection and more advanced screening of patients.

### Patient considerations

Patient adherence is a known barrier to successful outcomes [1]. In recent clinical trials patients that had 80% or more doses taken were still considered adherent and included in the final analysis [2,3]. We included random selection of doses taken in our model to simulate outcomes based on missing 20% of doses. We also investigated missing more doses in either the intensive or continuation phases of treatment to assess if an earlier or late drop in adherence has worse outcomes. Finally we used real world Medication Event Monitoring System (MEMS) data [41], to simulate observed patterns of missed doses and found that it followed a similar trend to our simulations of missing majority of doses in the continuation phase. For an earlier drop in adherence outcomes were much worse (86.0%). Missing doses closer together results in more patients failing treatment. This is often the case as treatment can be stopped and started depending on a clinician’s intervention due to side effect, illness or a patient’s access to care etc. Finding regimens that are more forgiving to these real world scenarios should be a priority.

Many patients with tuberculosis have reduced immune systems. This can be due to factors such as HIV or their disease severity. With this in mind we simulated a range of decreased immune system functions (25–75%). We found that in our model, drug treatment is able to treat a large number of patients successfully, however 6 months after treatment many patients start to relapse as their lowered immune systems is not able to fight off latent bacterial infection. These simulations suggest that patients with a lowered immune system may appear cured but are at higher risk of relapse after treatment.

### Important pharmacokinetic and pharmacodynamic model considerations

The assumed plasma clearance rate is a key factor in the concentration profile generated. It is common to find relatively large inter-person variations in drug metabolism and resulting plasma clearance rate. Model parameters were based on published experimental data of rifampin, isoniazid, pyrazinamide, and ethambutol. The clearance rate of these drugs was assumed to be independent of each other and of other drugs. Rifampin use can result in cytochrome P450 (*CYP*)-mediated drug-drug interactions with a wide set of drugs–in particular those metabolized by *CYP2B6*, *CYP3A4/5*, and *CYP2A6*. One common drug combination is the use of efavirenz, as part of HIV therapy, at the same time as rifampin for TB. This combination may increase the plasma clearance rate of rifampin by 15 to 35 percent [42]. Numerous other patient factors, such as renal and hepatic function, can affect the rate of metabolism and clearance of TB drugs. Further investigations of the impact of differences in TB drug net clearance among patients is warranted.

PK/PD dynamics were simplified and described minimal drug interaction effects. PK/PD interactions were only considered as related to drug concentration at the site of infection, where one drug might affect more efficacy in one area than another and therefore together increase or decrease each other’s efficacy. Simulations to show this effect are shown in S4 Fig, where the immune system response is responsible for killing in the extracellular space until drug is onboard. This simplified approach should be improved with the use of combination drug models that take into account the antagonistic or synergistic effects seen with multi-drug therapy. From drug treatment start RIF and INH have the largest kill on bacteria kill and fluctuate based on their respective penetration and therefore concentration is each compartment. PZA and EMB have minimal effect the first 60 days that they are onboard compared to RIF and INH.

We used a mechanistic bacteriostatic and bactericidal component in our model to mimic antibiotic classification where drugs can either inhibit the growth of bacteria, or increase their kill rate. Though the EC_50_ of bacteriostatic effects are much lower than bactericidal EC_50_’s and their contribution could seem minimal, our sensitivity analysis showed otherwise. Though the static influence is slight when concentrations fall below bactericidal but stay above static concentrations, growth inhibition still provides an effect. This is important in granuloma lesions where drug concentration can be limited, and a smaller effect is observed.

### Bacterial resistance considerations

The simulation model incorporated a basic representation of bacterial resistance with mutation rate parameters based on data from published *in-vitro* experiments. Naturally, many different types of mutations and other metabolic adaptations may occur. Bacterial mutations may also result in increased or reduced overall fitness, thus affecting rate of immune system killing of resistant bacteria strains. The mutation rate for isoniazid is an order of magnitude greater than for rifampin.

We investigated coverage of our multidrug therapies in intracellular and extracellular granulomas. We observed long periods of monotherapy (8 hours plus) and drug coverage in extracellular granulomas were estimated as 49%. Within this site of infection there was also no observed rifampin concentration greater than MIC, showing an area where resistance can therefor occur quickly. Some studies indicate bacteria which develop isoniazid resistance may have an increased probability of also achieving rifampin resistance, with a rate of 10–5 h^-1^[43]. We performed additional simulations looking at already INH resistant bacteria and the rate of then acquiring RIF resistance. Under full adherence conditions a patient is able to complete treatment without acquiring INH or multi-drug resistance. However in our simulations, when adherence falls to 60% a patient may develop multidrug resistant strains before the end of their treatment that will continue to grow and be selected for, S5 Fig.

### Model assumptions and suggested improvements

To implement the model, key assumptions often including simplification of the interplay between bacteria, host and drug were necessary. The model in its current phase of development does not include intracellular specific dynamics, which is an important consideration for bacterial growth and drug effects for total treatment duration as continuing research shows that drug MBC_90_ values are higher in some lesions compared to other and an shift in EC_50_ could be expected due to environment [44]. Similarly, the resistance model was narrowed down to mono-resistant strains and would greatly benefit from inclusion of mutation between different resistant strains to better understand the progression of multi-drug resistance and importantly how to optimize treatments to prevent this.

Though therapy outcomes included latent tuberculosis this was not related to dormant bacteria and there is no inclusion of slow replicating/dormant bacteria versus fast replicating bacteria. Therefore, the drug dynamics on these different states of bacterial growth are not captured, including a mechanistic approach to describing the reduction in killing rate which in our model is estimated from raw data for specific time ranges. This decline may be due to several reasons, including growth of resistant sub-populations of bacteria, reduction in absolute remaining number of bacteria, and incomplete mixing of active drugs and bacteria thus limiting its therapeutic effect. In addition to these cellular considerations, our model also did not take into account actual tissue mass, protein binding or physical lesion size and distance from vascularity. A physiological based pharmacokinetic (PBPK) approach could offer insight into these mechanisms, for e.g. Edginton and colleagues were able to build a whole-body PBPK model that described penetration of moxifloxacin into macrophages using colorectal biopsies [45].

The model was limited to HRZE to first assess its outcomes to clinical literature, but new drugs and drug therapies should be added to keep the model up to date with future regimens. Only acetylator status was added as a categorical covariate, but the population PK models used had additional covariates on sex, HIV status to name a few that could be incorporated in the pharmacokinetic models to look at individuals at risk of low exposure and poor outcomes.

Finally, the model was parameterized based on a wide array of available literature and would benefit from further experiments that may be able to refine or validate parameter values used and consolidate the model complexity with similar sources. The advantage of this systems model approach is that the experiments could be diverse including mouse, *in-vitro* hollow fiber system experiments, physiologically-based pharmacokinetic results and or ongoing clinical trial data to clarify and update parameters and build on the structural model. Additionally a limitation of our model is the fairly complex parameter space with low certainty about the variance-covariance structure, which is inherent when applying parametric distributions. Access to reliable parameter correlations and potentially nonparametric distributions would improve the performance of the model.

### Clinical relevance and applications

Numerous large-scale clinical trials are under way to evaluate new drugs and drug therapy combinations to treat pulmonary TB infection [46–48]. The simulation model presented in this study could be used to help focus such clinical trial investments on those new drug combinations predicted to offer greatest likelihood of having performance that is better than existing standard TB therapy. The model has the great advantage of focusing limited resources and speeding up the trial planning process. By fine-tuning drug dosing parameters and adapting drug therapy to additional patient populations, models like these can help predict study success and eliminate insufficient studies.

## Materials and methods

The model building process did not include studies with human participants, animals or field work performed by any of the authors and did not require ethics approval.

### Bacterial infection and growth

**Fig 14:**
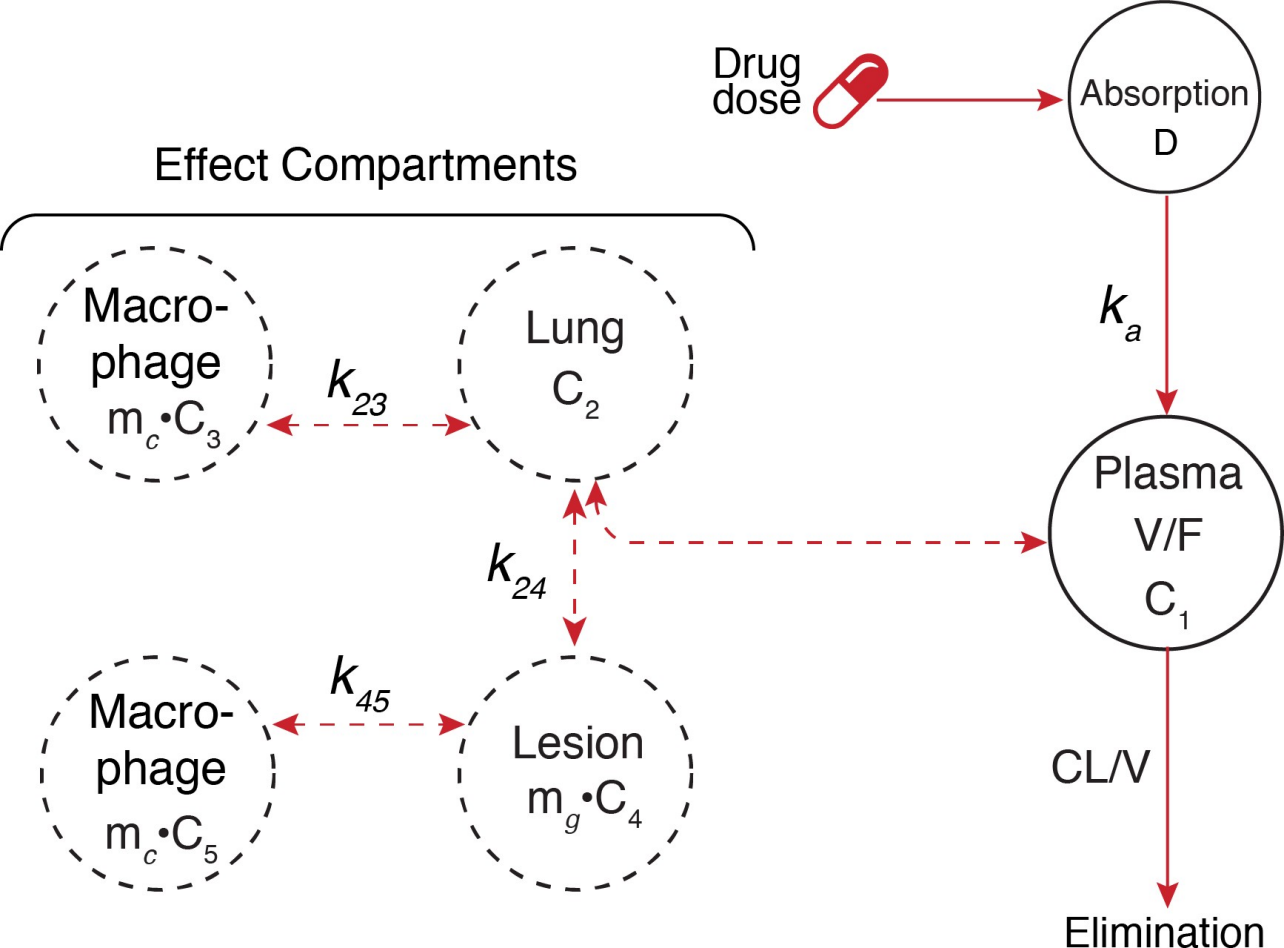
Overview of disease model. Description of key aspects of the disease model. Bacterial infection occurs by aerosolized bacteria reaching the lungs. The bacteria remain in lung tissue, and may be absorbed into macrophages. With long-standing disease granulomatous lesions may form, which in-part may work to contain the bacteria, but also may limit effectiveness of drug therapy and thus form a partial reservoir of bacteria. Bacterial growth, killing, mutation, and drug effects are present for each model compartment. Dotted lines represent movement of immune cells and solid lines represent movement of bacteria.

Pulmonary infection is assumed to be transmitted through aerosolized droplets containing *M*. *tuberculosis* bacteria. Once in the lung, characterized by our model as extracellular space, bacteria penetrate into alveolar macrophages, where they proceed to grow according to rates characteristic for pulmonary tuberculosis. In line with the originally validated model from Marino et al., 2004 uncontrolled infection with *M*. *tuberculosis* eventually results in sustained high bacterial density, both outside and inside of macrophages, often reaching maximum levels of 10^7^ to 10^9^ CFU/mL in the lung compartments, after 100 to 200 days from initial infection. Sustained infection may trigger a complex host immune-bacteria reaction leading to formation of encapsulation of infected tissues. The inflamed tissue forms nodules, and may grow to become larger granulatomous lesions. Here the immune system is actively sequestering the infection into lesions to help prevent further spread of bacteria. If no immune system was on board the infection would grow further without stopping, S6 Fig. A major contribution of the immune effect in our model is to simulate the environment of lung lesions and provide the necessary barrier to drug diffusion that we expect from macrophages and other immune cells. The lesions can be considered a distinct compartment, and may contain substantial concentrations of bacteria. The integrated disease model and immune model compartments are illustrated in Fig 14 [30].

Net bacterial growth was defined as the sum of inherent bacterial growth and death rates, adjusted for effects from pharmacodynamics, immune system, and bacterial mutation. The general form of growth of bacteria population *B*_*i*_ was described by Eq (1),

$$\frac{dB_{i}}{dt}=B_{i}*\left(g_{i}^{max}*f_{i}*G_{i}+K_{i}+d_{i}\right)+\Delta B_{i}^{im}+\Delta B_{i}^{mu}$$ (1)

where $g_{i}^{max}$ was maximum hourly growth rate (h^-1^), *f*_*i*_ was relative fitness level {0…1}, *G*_*i*_ was natural bacterial growth adjusted for drug bacteriostatic effects, and *K*_*i*_ is the drug bactericidal effect, $\Delta B_{i}^{im}$ was the immune system effect, and *d*_*i*_ was the natural death rate of bacteria population *i*. The term $\Delta B_{i}^{mu}$ represented the effect due to mutation from wild-type to mono-resistant strains. Additionally, for bacteria inside macrophages, net growth rate was limited by maximum bacteria capacity inside a macrophage, per immune system model by Marino & Kirschner [30]. The growth limitating factor applied to bacteria inside macrophages was described by Eq (2),

$$\left(1-\frac{B_{I}^{m}}{B_{I}^{m}+{\left(NM_{I}\right)}^{m}}\right)$$ (2)

where *B*_*I*_ was the number of bacteria inside macrophages, *N* was the maximum number of bacteria inside macrophages, *M*_*I*_ was the number of infected macrophages, and *m* a constant.

### Immune system response

The immune system model portion was based on an approach developed and validated by Marino & Kirschner, originating from an extensive line of work by the Kirschner group. The immune system was defined by a system of differential equations, representing different constituent cell types—macrophages, cytokines, T cells, and dendritic cells–driving cell-mediated immune system response to bacteria in pulmonary TB infection. The immune system model incorporated dynamics of intra- vs. extracellular bacteria, cytokine signaling, and T-cell response in lung and lymph node. The original immune system models were extended to support multiple mutated bacterial strains, and additional compartments to represent lung granuloma lesions [30]. Typical values for the immune system and bacterial model parameters were based on data provided, except as described in Table T1, in the S1 Text that contains all immune system components. Patient-specific parameter values were generated using inter-individual variance, as well as stochastic errors applied at each time step. Initial values for model variables were based on [30,49] for TB infection, as summarized in Table T2, in S1 Text. This allowed simulated patients to have an active case of TB infection at the start of treatment.

### Patient drug therapy adherence

High patient adherence status is critical to achieve effective outcome of long-term TB therapy. In the simulation model, patient-level adherence is determined at daily level based on an integrated adherence probability module. The adherence module generates single- or multi-day non-adherence events using a normal distribution, or a discrete probability distribution Γ(*p*,d) for probability *p* of non-adherence event of length *d* days, and where each patient has a unique long-term adherence profile. Note: for validation of the standard TB therapy simulation model, patient drug therapy adherence was set to 1.

### Pharmacokinetics model

A population pharmacokinetic simulation model including rifampin, isoniazid, pyrazinamide, and ethambutol was developed and configured with previously validated parameters and published model structures and parameters. These parameter values were based on data from published research studies with references shown with final pharmacokinetic parameters in Table 2 and a bibliography of parameters and models with their respective reference can be found in S3 Text.

**Table 2:** Pharmacokinetics core model parameter values.

Drug	Absorption rate, Ka (h^-1^)	Clearance rate, CL/F (L/h)	Apparent vol. of distribution V/F (L)	Elimination rate, Ke (h^-1^)	Other factors
Rifampin [14]	1.81 (48%)	8.1 (37%)	49.6 (24%)	0.18 (34%)	Auto-induction factor 2.02, over 17 days
Isoniazid [19]	2.21 (76%)	17.8 (20%)	75.0 (17%)	0.22 (18%)	Clearance rate multiplier for “fast acetylators” 2.50
Pyrazinamide [50]	3.42 (62%)	4.3 (16%)	39.4 (9%)	0.104 (23%)	
Ethambutol [51]	0.57 (28%)	61.1 (24%)	230 (33%)	0.129 (30%)	

Note: value in parenthesis is %CV due to inter-individual variability.

Drug concentration in plasma and extracellular alveolar fluid was based on a one-compartment model, with first order absorption and linear elimination. Drug concentration inside macrophages and inside granuloma were modeled as separate effect compartments, with factors for attenuation, accumulation, and time delay in concentration relative to plasma. In this simulation, granuloma was modeled as a single virtual compartment, thus representing a composite of various such lesions in the lung, likely to vary greatly in size, caseation, fibrosis, and vascular access. The overall pharmacokinetic model is depicted in Fig 1. The effect compartment parameters were assumed to be independent of drug concentration, and fixed over time. Auto-induction effects on clearance were added where applicable. Auto-induction effects were simplified to prorated based on actual number of days of drug administration during preceding period for e.g. rifampin applied an auto-induction multiplier of 2.02 after 17 days to simplify the model structure and resulting computation time. Parameter values for individual patients were simulated based on defined typical values and applying inter-individual variance, as well as stochastic errors for each time increment.

#### Rifampin

Rifampin will enter TB cell cytoplasm and is believed to interfere with bacterial DNA replication. Concentration of rifampin inside macrophages (“intracellular”) is believed to be higher than outside macrophages (“extracellular”), and may have a residual concentration profile creating an extended antibiotic effect [12]. The precise intracellular concentration dynamics is not fully understood, and likely is affected by changes in resistance and metabolic state of bacteria. Earlier published simulation of intracellular rifampin concentration was done using a 3-compartment pharmacokinetic model [49,52]. For this study, effect compartment parameters were set so intracellular rifampin concentration was at three to four times extracellular concentration. Intracellular rifampin concentration was set to decay at lower rate vs. in the extracellular space. Auto-induction was modeled with a steady-state multiplier of approximately two times vs. base line, increasing linearly over 17 days, thus resulting in twice the rate of elimination [14,53].

#### Isoniazid

For the isoniazid pharmacokinetic model, the patient population was divided into two categories based on rate of drug metabolism—so called *fast* vs. *slow* acetylators. Fast acetylators are estimated to have approximately twice the clearance rate of slow acetylators. The proportion of fast vs. slow acetylators varies for different ethnic and geographic populations [19,50,54]. For the base simulation, it was estimated fast acetylators represented approximately half of the total patient population (48%) [55].

#### Pyrazinamide

The pyrazinamide effect compartment parameters assumed relatively similar drug concentration for intra- and extracellular compartments [56]. No auto-induction effects were assumed for pyrazinamide.

#### Ethambutol

Ethambutol is assumed to reach similar concentration outside vs. inside of macrophages. It has been suggested ethambutol may have synergistic effect with rifampin and isoniazid, due to the ability of ethambutol to reduce integrity of the bacterial cell wall to allow increased concentration of other drugs to enter the bacteria [57]. The current simulation model did not include any drug-drug synergy effects.

#### Mathematical model for pharmacokinetics

Plasma drug concentration *C*(*t*) for drug *n* is derived by calculating the quantity of drug in plasma *A*(*t*) following the drug dosing schedule *D*(*t*), with adherence *a(t)* per patient. The model is based on a single-compartment system with first-order absorption and elimination, based on Eqs (3) and (4).

$$\frac{dA}{dt}=k_{a}*D(t)*a(t)-k_{e}(t)*A(t)$$ (3)

$$\frac{dD}{dt}=-k_{a}*D(t)*a(t)$$ (4)

where *A*(*t*)≥0 and *D*(*t*)≥0, and *a(t)* was {0..1}. Parameter *k*_*a*_ was the rate of oral absorption of drug *n*, *k*_*e*_(*t*) is derived by total clearance (*CL*/*F*) divided by apparent volume of distribution (*V*/*F*) for drug *n*; and *A*(0) and *D*(0) were set to 0. Patient adherence *a(t)* is defined at daily level and applied equally for all drugs on that day. The concentration *C*(*t*) is calculated independently for each drug, and derived as *A*(*t*) divided by *V*/*F*. The coefficient of elimination *k*_*e*_(*t*) for any drug *n* includes factors for auto-induction and acetylation rate per patient, as shown in Eq (5). Increased clearance rate due to auto-induction is approximated with a linear function from $k_{e}^{min}$ at day 0 to steady-state $k_{e}^{max}$, reached after *t*^*max*^ days.

$$k_{e}(t)=k_{e}^{min}*(1+(k_{e}^{max}/k_{e}^{min}-1)*t/t^{max}$$ (5)

for *t*_0_<*t*<*d*, and $k_{e}(t)=k_{e}^{max}$, when *t*>*t*^*max*^, and prorated for actual number of days of drug administration during the preceding time period to account for a lack of adherence if included. This auto-induction effect takes place relatively quickly within the context of total treatment days. The mathematical structure differed from the original semi-mechanistic model proposed by Smythe et al 2012 [14], but the improved computation speeds and similar PK output simulations were found suitable account for these17 days.

**Fig 15:**
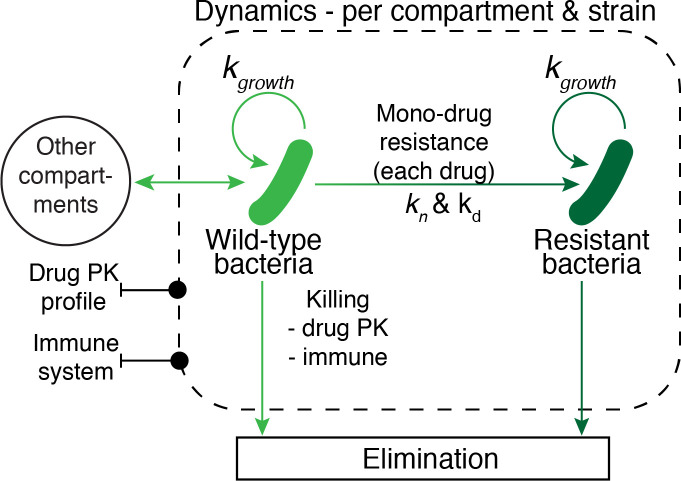
Overview of pharmacokinetic model. Description of the integrated pharmacokinetic (PK) model. Drug concentration in plasma and extracellular alveolar fluid was based on a one-compartment model, with first order absorption and linear elimination. Drug concentration inside macrophages and inside granuloma were modeled as separate effect compartments.

Concentration of drug and active metabolites in the intracellular compartment, is modeled using an effect compartment model which describes a time delay in drug transfer between extra and intra cellular space. This process is described both in lung and lesion compartments, Fig 15. The parameters describing uptake into intracellular space were determined from macrophage uptake studies, see S3 Text. We simulated drug distribution from the plasma directly to macrophages and lesions due the experiments used to estimate the parameters and therefore equalize our “lung” to the plasma to allow correct drug levels in the highest bacterial burden compartments of interest. From the lung compartment drug can move into macrophages and is defined as intracellular concentration, or into granuloma lesions and defined as extracellular granuloma concentration. From the extracellular granuloma space, drug can distribute into macrophages inside granulomas and are then defined as intracellular granuloma concentration. The absolute quantity of drug in these compartment types is assumed to be several orders of magnitude lower than in plasma, therefore having minimal effect on the plasma compartment concentrations. Effect compartment factors are assumed to be independent of plasma and compartment drug concentration, and fixed over time. The effect compartment dynamic is described by Eq (6).

$$\frac{dC_{e}}{dt}=m_{eq}*k_{eq}^{\Delta }(C_{p}-C_{e})$$ (6)

where *C*_*p*_ is concentration in plasma, *C*_*e*_ is concentration in effect compartment, *m*_*eq*_ is concentration multiplication factor for effect compartment, and $k_{eq}^{\Delta }$ is the equalization constant, $k_{eq}^{\Delta }=k_{eq}^{+}$ when *C*_*p*_ is increasing, and $k_{eq}^{\Delta }=k_{eq}^{-}$ when *C*_*p*_ is declining to account for drug entering and exiting the compartment. Larger values for *k*_*eq*_ will result in faster equalization of concentration ratios between compartments. Selected values for effect compartment parameters are summarized in Table 3 and their bibliography of sources can be found in S3 Text in the pharmacokinetic section of each drug.

**Table 3:** Pharmacokinetics effect compartment parameter values.

Drug	Intracellular (macrophage) multiplier, *m*_*eq*_	Intracellular time constant (h^-1^), $k_{eq}^{\Delta }$	Granuloma multiplier, *m*_*eq*_	Granuloma time constants (h^-1^), $k_{eq}^{\Delta }$
Rifampin	3.7	In: 0.1 Out: 1.0	0.15	In: 5.0 Out: 5.0
Isoniazid	0.95	In: 0.1 Out: 0.9	0.28	In: 0.1 Out: 1.0
Pyrazinamide	0.83	In: 0.1 Out: 0.9	1.25	In: 0.1 Out: 1.0
Ethambutol	1.5	In: 0.1 Out: 0.9	0.45	In: 0.1 Out: 1.1

### Pharmacodynamics

**Fig 16:**
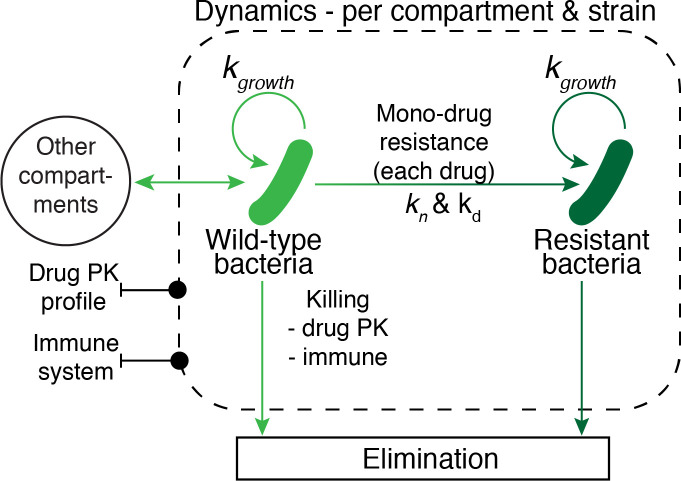
Overview of pharmacodynamic model. Description of the integrated pharmacodynamic (PD) model for each compartment of the simulation model, incorporating bacterial growth, death, exchange with other compartments, bacterial drug resistance, and killing by immune system effects and active drug concentrations.

Bacterial survival and growth is influenced by bacteriostatic and bactericidal effects from all drugs acting on any given bacteria population in each specific compartment. The effects of bacterial mutation and development of resistance are detailed in its relevant section. Using principles defined by Gumbo et la., 2004, drug concentration for each drug *n*, per compartment, may have both bactericidal *K*_*i*_ and bacteriostatic *G*_*i*_ effects on each bacteria population *i* [58]. The total pharmacodynamic effects of each drug are calculated based on the actual drug concentration at any given point in time, relative to the *EC*_50_ level, and integrating over time. The parameter *EC*_50_ represents the concentration where 50 percent of a bacteria population is affected. The simulation model incorporates two *EC*_50_ values per drug, one for bactericidal effect, and one for bacteriostatic effect. The same *EC*_50_ value is applied for both intra- and extracellular bacteria, and the parameter is assumed to remain fixed over time. The *EC*_50_ parameter values are based on research studies using the Hollow Fiber Model [57–60], as summarized in Table 4, bibliography of sources for these parameters can be found in S3 Text. The combined growth dynamics of each bacteria population within each model compartment is illustrated in Fig 16 and effect compartment parameters are summarized in Table 5.

**Table 4:** Pharmacodynamics model parameter values.

Drug	Bacteriostatic effect		Bactericidal effect	
	EC_50_ (mg/L)	Hill factor	EC_50_ (mg/L)	Hill factor
Rifampin	1.5	0.36	5.0	0.79
Isoniazid	0.1	1.05	0.4	1.05
Pyrazinamide	50.0	1.21	100.0	1.21
Ethambutol	1.0	1.0	4.0	1.0

**Table 5:** Effect compartment parameter values.

	Granuloma (k_14_)		Macrophage (k_23_, k_45_)	
Drug	T^in^_1/2_ (h)	T^out^_1/2_ (h)	Rise factor	Decay factor
Rifampin	5	5	1.0	0.3
Isoniazid	0.1	1.1	1.0	0.9
Pyrazinamide	0.1	1.1	1.0	0.9
Ethambutol	0.1	1.1	1.0	0.9

* Decay factor defines decay characteristics for drug concentration elimination from macrophages, where a value 0.0 is no decline, and value 1.0 is immediate decline due to falling concentration outside of macrophage. The rise factor is set to 1.0 for all drugs, reflecting immediate uptake of increased drug concentration from outside to inside of macrophages.

#### Bactericidal effects

Total bactericidal effect on bacteria population *i* was described by Eq (7),

$$K_{i}=\sum_{n=1}^{N}k_{n,i}^{max}(t)*p_{n,i}(t)\frac{1}{1+{\left(C_{n}^{50k}/C_{n}\right)}^{\alpha_{n}^{k}}}$$ (7)

for all drugs *n*{1..N} acting on bacteria population *i*, where *N* was the number of drugs, $k_{n,i}^{max}(t)$ was maximum kill rate, for drug *n* on population *i* at time *t*. Max kill rate may vary per time period after therapy start, i.e., day 0–2, 3–14, and 15+. The maximum kill rate is defined as greatest bacterial killing possible to achieve at very high drug concentrations. The parameter is expressed in unit of *h*^−1^ used in the standard expression for bacterial growth and killing, *dB*/*dt* = (*g*−*K*_*i*_)*B(*t*), where *B(t)* is the bacterial population at time *t* in hours, and *K*_*i*_ is hourly kill rate, and *g* represents growth rate factors as described further below. Research studies often report maximum bacterial kill rate in terms of *log*_10_ kill per day. The *log*_10_ kill per day, (*E*_max_)*log*_10_, can be transformed to hourly kill rate, *K*_*i*_, using the expression *K*_*i*_ = (*E*_*max*_)*log*_10_*ln(10)/24. The parameter *p*_*n*,*i*_(*t*) has values {0…1} to incorporate effectiveness of killing by drug *n* given current growth rate of bacteria population *i* at time *t*, as some drugs are thought to have higher or lower ability to kill slow-growing, aka "persisting,” bacteria." The factor $\alpha_{n}^{k}$ is the Hill coefficient of sigmoidicity for bacterial killing of drug *n*, and $C_{n}^{50k}$ is the median effect concentration for bacterial killing for drug *n* and finally *C*_*n*_ is the concentration of drug_*n*_ as described by our longitudinal PK model. Bacterial killing is assumed to occur independently by each drug, and total bactericidal effect on a bacteria population was additive.

#### Bacteriostatic effects

Total bacteriostatic effect on bacteria population *i* is described by Eq (8),

$$G_{i}=\prod_{n=1}^{N}\left(1-\frac{1}{1+{\left(C_{n}^{50g}/C_{n}\right)}^{\alpha_{n}^{g}}}\right)$$ (8)

for all drugs *n*{1..N} acting on bacteria population *i*, where *N* was number of drugs, $\alpha_{n}^{g}$ is the Hill coefficient of sigmoidicity for bacterial growth inhibition of drug *n*, and $C_{n}^{50g}$ is the median effect concentration for bacterial growth inhibition by drug *n* and *C*_*n*_ is the concentration of drug_*n*_ as described by our longitudinal PK model. Bacterial growth inhibition is assumed to occur incrementally among drugs, and total bacteriostatic effect is calculated additively across all active drugs.

#### Bacterial kill rates

The bacterial kill rate for each drug depends on numerous factors, including intra- and extracellular drug concentrations, drug therapy duration, bacterial growth characteristics, bacterial resistance development, and local microenvironment characteristics such as pH. Many *in-vitro* studies have reported “net kill rates” of commonly used TB drugs decline over time, see S3 Text. The decline in drug effectiveness over time is modeled with time-dependent effective bacterial “kill rates”; with time structured into three periods: day 0–2, day 3–14, and day 15+, measured from day of therapy start. Maximum effective bacterial kill rates are estimated based on data from published research reports: for rifampin [36,49,57,60–64]; for isoniazid [57,62,65]; for pyrazinamide [50,56,62,65,66], and for ethambutol [25,35,57,67], as summarized in Table 6.

**Table 6:** Effective bacterial kill rates for tuberculosis drugs (outside/inside macrophages).

Drug	Kill rate day 0–2 (h^-1^)	Kill rate day 3–14 (h^-1^)	Kill rate day 15+ (h^-1^)
Rifampin	0.5 / 0.5	0.4 / 0.4	0.4 / 0.4
Isoniazid	0.4 / 0.05	0.2 / 0.02	0.1 / 0.01
Pyrazinamide	0.0 / 0.0	0.02 / 0.04	0.02 / 0.04
Ethambutol	0.05 / 0.05	0.04 / 0.04	0.03 / 0.03

Note: the above parameter for “kill rate per hour,” is different from the “Δ Log CFU/mL per day” kill rate metric sometimes reported in experimental studies.

#### Bacterial drug resistance

Bacterial drug resistance may develop during tuberculosis treatment with antibacterial agents, in particular following plasma drug concentrations at sub-therapeutic levels. The pharmacodynamic and immune system models were extended to incorporate bacterial mono-resistance to each individual drug, and bacterial exchange of resistant bacteria between intra- and extra- macrophage compartments. The probability of multi-resistant mutations typically is several orders of magnitude lower–as long as the patient population does not have pre-existring resistance to drugs used.

Development of bacterial drug resistance is modeled using distinct sub populations, i.e., four mono drug-resistant bacteria strains for each compartment. Resistance to specific drugs occurs naturally, and resistant strains are selected for when drug monotherapy occurs increasing overall resistance in that population. To model this we include a natural resistant rate due to random mutation from wild-type to become drug-resistant, per Eq (9) and a drug pressure rate that increases the number of resistant bacteria when drug is on board.

$$\Delta B_{i}^{mu}=B_{w}*\frac{dB_{w}}{dt}*r_{w,i}$$ (9)

where the factor $B_{w}*\frac{dB_{w}}{dt}$ is net growth of wild-type bacteria in the same compartment, *r*_*w*,*i*_ is mutation rate of wild type bacteria to the drug resistant bacteria population *i*, in h^-1^, and where $\Delta B_{i}^{mu}\geq 0$. The number of mutated bacteria is derived based on the base growth rate of wild-type bacteria before subtracting bactericidal effects of drugs and immune system, and natural death rate. The net growth of wild-type bacteria thereafter is reduced accordingly. The initial population of resistant extracellular bacteria is set as a very small ratio of the extracellular bacterial inoculate, e.g., (1∙10^−9^). Parameters values for mutation rate of wild-type to different drug mono-resistant strains, *r*_*w*,*i*_, are based on data from published studies [43,68,69], as summarized in Table 7.

**Table 7:** Resistant mutation rates.

Drug	Natural rate of drug resistance (*kn*)	Mutation rate during drug therapy (per generation*) (*kd*)
Rifampin	1∙10^−9^	8.9∙10^−9^
Isoniazid	1∙10^−9^	2.2∙10^−8^
Pyrazinamide	1∙10^−9^	1.1∙10^−5^
Ethambutol	1∙10^−6^	5.5∙10^−6^

* Note: bacterial mutation rates per generation. Average replication time for *M*. *tuberculosis* assumed to be 24 hrs. Hourly mutation rate is 1/24^th^ of the per-generation mutation rate.

### Population modeling and patient outcomes

Patient individual values are generated for parameters in each sub model, including initial bacterial infection, pharmacokinetic, pharmacodynamic, immune system, and bacterial drug resistance. There are assumed to be no systematic changes in pharmacokinetic parameters during the treatment period, besides auto-induction effects. Patient variability for other parameters, such as immune system effects and pharmacodynamics, are based on a log-normal distribution, unless the referenced experiment stated otherwise. To replicate inter-occasional variability small random error perturbations are applied to the pharmacokinetic parameters per time step of the simulation, using a uniform distribution. This represents the real world scenario where a patient’s clearance might be slightly different between doses [70]. Key inter-individual variability and time error methods and parameter values are summarized in Table 8.

**Table 8:** Standard deviation of population patient parameter values.

Drug	Ka	Ke	V	EC_50_
Rifampin	0.66	0.31	0.25	0.10
Isoniazid	0.58	0.20	0.11	0.10
Pyrazinamide	0.52	0.14	0.10	0.10
Ethambutol	0.27	0.08	0.40	0.10

The simulation program is set up to monitor infection level per patient, per compartment, at each time point during the simulation, and this is used to derive a disease status, defined as either *TB infection*, *Latent TB*, or *Cleared TB*. A patient is considered to have a Cleared infection status if *total* bacterial load is measured to be below a certain threshold level, such as 1 CFU/mL. Rules for classification of different therapy outcomes are summarized in Table 1. Relapse is defined as patients that have TB infection still present.

Patient therapy is considered successful when *total* bacterial concentration, i.e., wild type and various resistant strains, present outside of granuloma is less than 1 on the last day of pharmaceutical therapy and the patient infection set to 0. Simulation outcomes are found to be relatively consistent across a range of values from 1 to 100 CFU/mL. Any residual concentration of bacteria inside granuloma may be used to distinguish between a possible Latent TB infection or Cleared TB, this aspect of the simulation model may be further explored in follow-on research work.

### Numerical methods and simulation calculation flow

Key steps in simulation calculation flow were as follows,

1. Initialize overall model and simulation parameters: drug PK/PD parameters, patient adherence, drug therapy timing and dosing, immune system parameters, bacterial drug resistance mutation rates, and simulation threshold levels and other configurations.
2. Generate effective dosing vectors per day for each drug and patient, based on selected drug therapy schedule, and simulated adherence profile per patient.
3. Calculate pharmacokinetics using the Euler method, with 100 steps per time period (hour), to create a vector of hourly concentration level, per drug and per compartment.
4. Calculate immune system and pharmacodynamic effects at hourly level, using a fourth-order Runge-Kutta approximation to solve system of differential equations, with strict non-negative conditions for all bacteria populations, immune cell types, and cytokines.
5. Transform bacterial infection and immune system results from hourly to daily averages.
6. Censor very low infection levels, i.e., if sum of all bacteria per time period is less than 1 CFU/mL–then infection is considered eliminated.
7. Determine patient infection status and therapy outcome, per rules described above.
8. Repeat calculation steps (2) to (7) for each patient in a simulated population of N patients, from time of initial infection for the duration of simulation (up to 1000 days).
9. Use bootstrap method to generate M populations of N patients each
10. Generate statistics for infection and outcome variables across M different populations

Numerical computations, including bootstrap processing, were implemented with a program written in GNU C++ (64-bit), using OpenMP libraries to optimize multi-core calculations. Reporting and visualization of simulation results were performed using R. The simulation model was tested using numerous different drug therapy and population scenarios, with up to 10,000 patients and 1,000 days. Typical execution times, using a mid-sized Intel quad-core computer running at 2.6 GHz, for a simulation involving 1000 patients, with 1000 bootstrap populations, over 500 days, with standard 4-drug TB therapy, bacterial drug resistance, and granuloma dynamics, was approximately 60 seconds. The computer code used for simulation and results visualization is available on https://github.com/saviclab/TBsim.

In addition to the core numerical tool, a wrapper package for R interfacing with the simulation tool was also developed to facilitate easier customization of simulations and generation of reports and plots. The R package is made available under an open source license at https://github.com/saviclab/TBsim. In addition, a web application was developed that exposes the functionality of the simulation tool in an easier-to-use user interface, based on the Shiny web application framework. The tool is freely available for use at http://www.saviclab.org/systems-tb/ and includes fast “Single Patient” simulations, either typical or randomly generated, a “Population” panel that simulates relapse percentage in a population over time and finally a “Drug” panel where new drugs can be entered and evaluated.

## Supporting information

S1 Fig Individual patient drug concentration profiles show large variations. Intracellular concentration of rifampin for ten random patients during initial week of drug therapy. Grey lines represent individual patients while the red line represents the median for this group. Large variance is observed, for example, maximum concentration varies more than factor 2x among these patients.
(TIF)

S2 Fig Summary drug concentration profiles per drug. Population average concentration of each drug as calculated for extra- (blue) and intracellular (red) compartment, for initial two weeks of drug therapy. This simulation assumes standard therapy and 100% patient adherence.
(TIF)

S3 Fig Sensitivity analysis of immune and initialization parameters. Parameters were scaled by factor 0.1, 0.5, 2 and 10, and the final change in percentage of the population with TB (mean 5% under normal conditions) recorded to measure individual parameter impact on TB outcome.
(TIF)

S4 Fig Bacterial dynamics of simulated TB infection in different compartments. (TIF)

S5 Fig Progression of multi-drug resistance with different adherence settings in intracellular granuloma. (TIF)

S6 Fig Bacterial dynamics of simulated TB infection with and without immune system on board. (TIF)

S1 Text Immune System Components. (PDF)

S2 Text Clinical Trial Data. (PDF)

S3 Text Model parameters and sources. (PDF)
