# Supplementary figures and images for: Mathematical model and tool to explore shorter multi-drug therapy options for active pulmonary tuberculosis
Source: PLoS Comput Biol. 2020 Aug 18;16(8):e1008107. doi: 10.1371/journal.pcbi.1008107 (PMC7480878; doi:10.1371/journal.pcbi.1008107)

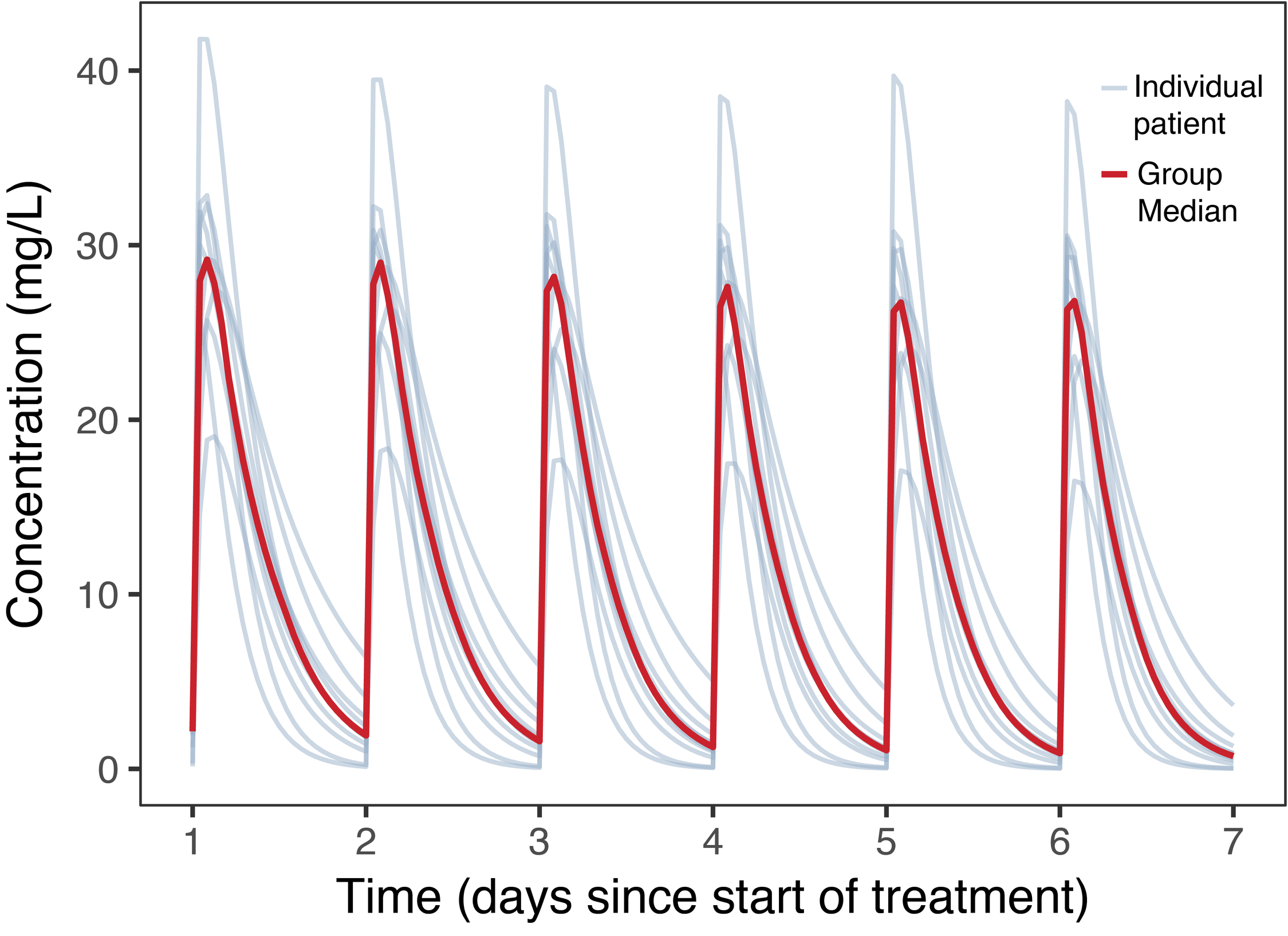

Supplement: S1 Fig — Intracellular concentration of rifampin for ten random patients during initial week of drug therapy. Grey lines represent individual patients while the red line represents the median for this group. Large variance is observed, for example, maximum concentration varies more than factor 2x among these patients. (TIF) [file pcbi.1008107.s001.tif]

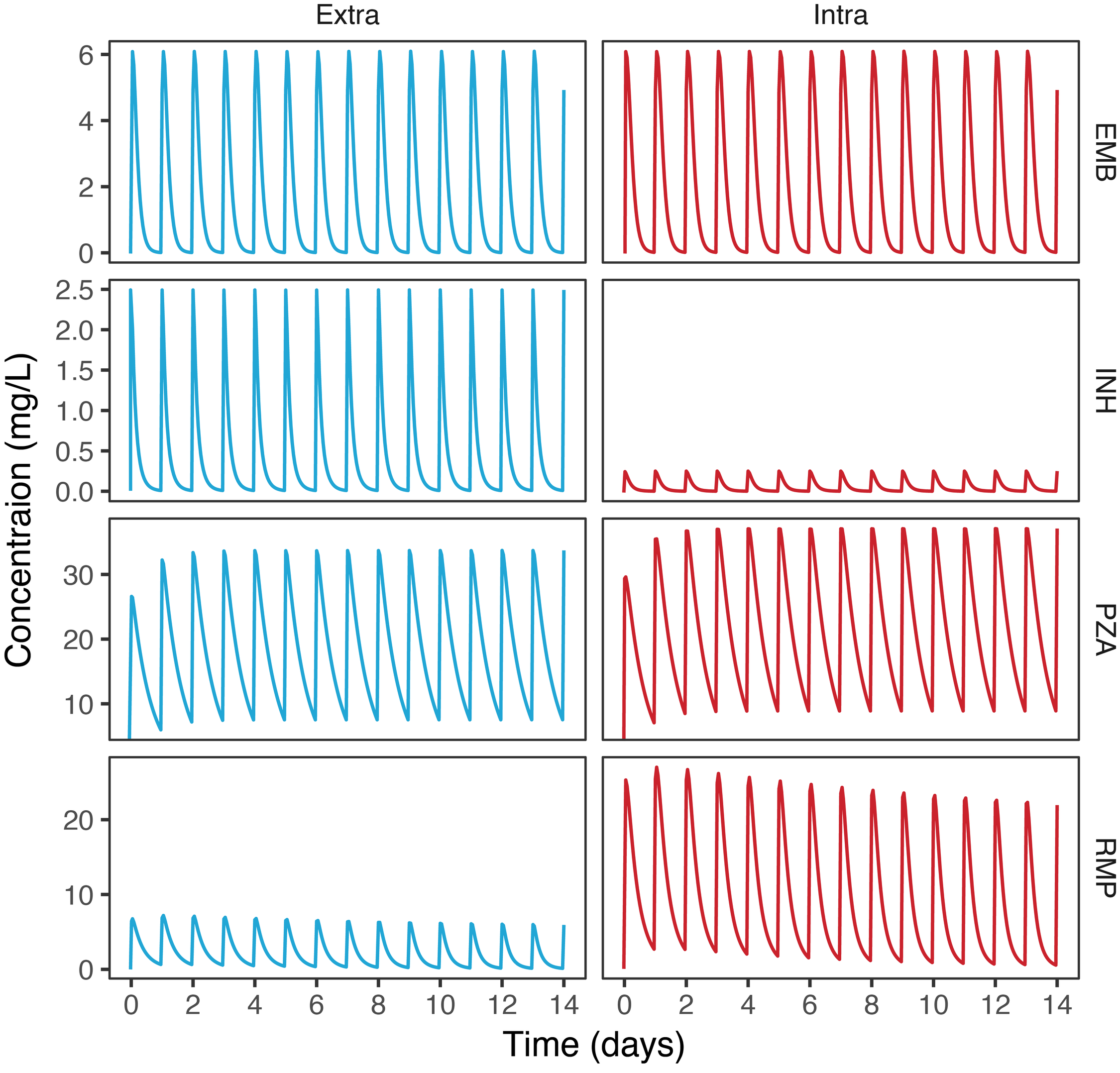

Supplement: S2 Fig — Population average concentration of each drug as calculated for extra- (blue) and intracellular (red) compartment, for initial two weeks of drug therapy. This simulation assumes standard therapy and 100% patient adherence. (TIF) [file pcbi.1008107.s002.tif]

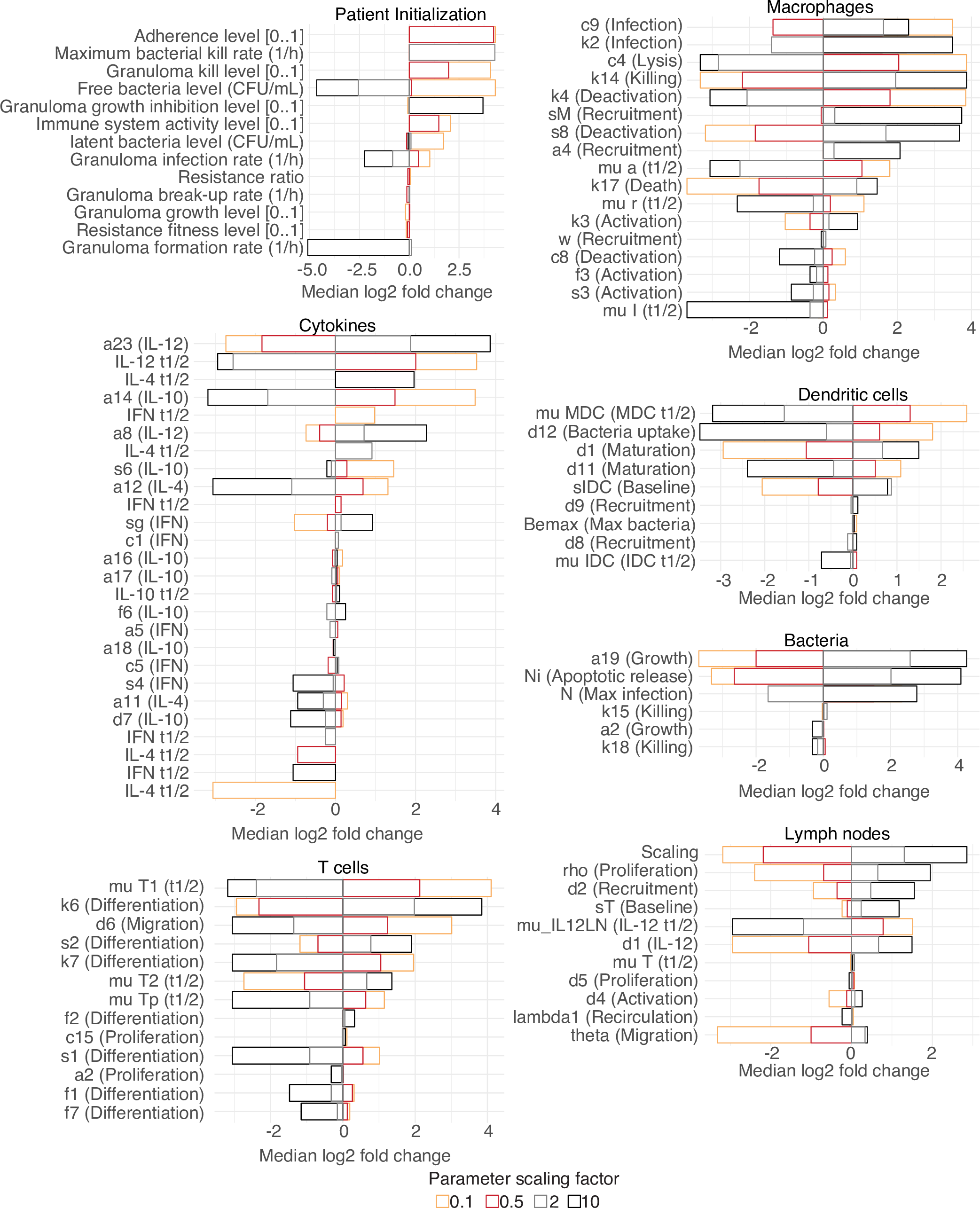

Supplement: S3 Fig — Parameters were scaled by factor 0.1, 0.5, 2 and 10, and the final change in percentage of the population with TB (mean 5% under normal conditions) recorded to measure individual parameter impact on TB outcome. (TIF) [file pcbi.1008107.s003.tif]

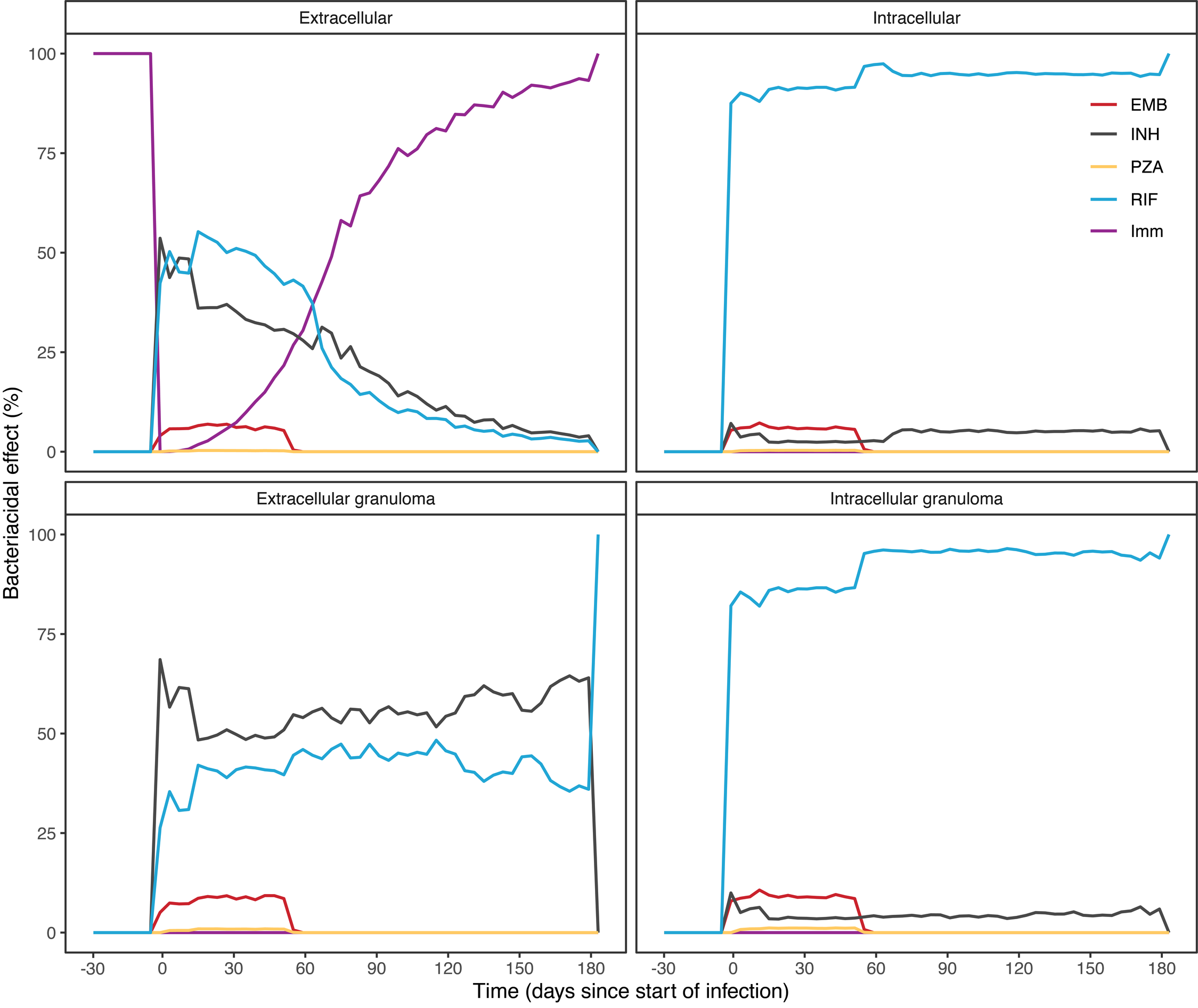

Supplement: S4 Fig — (TIF) [file pcbi.1008107.s004.tif]

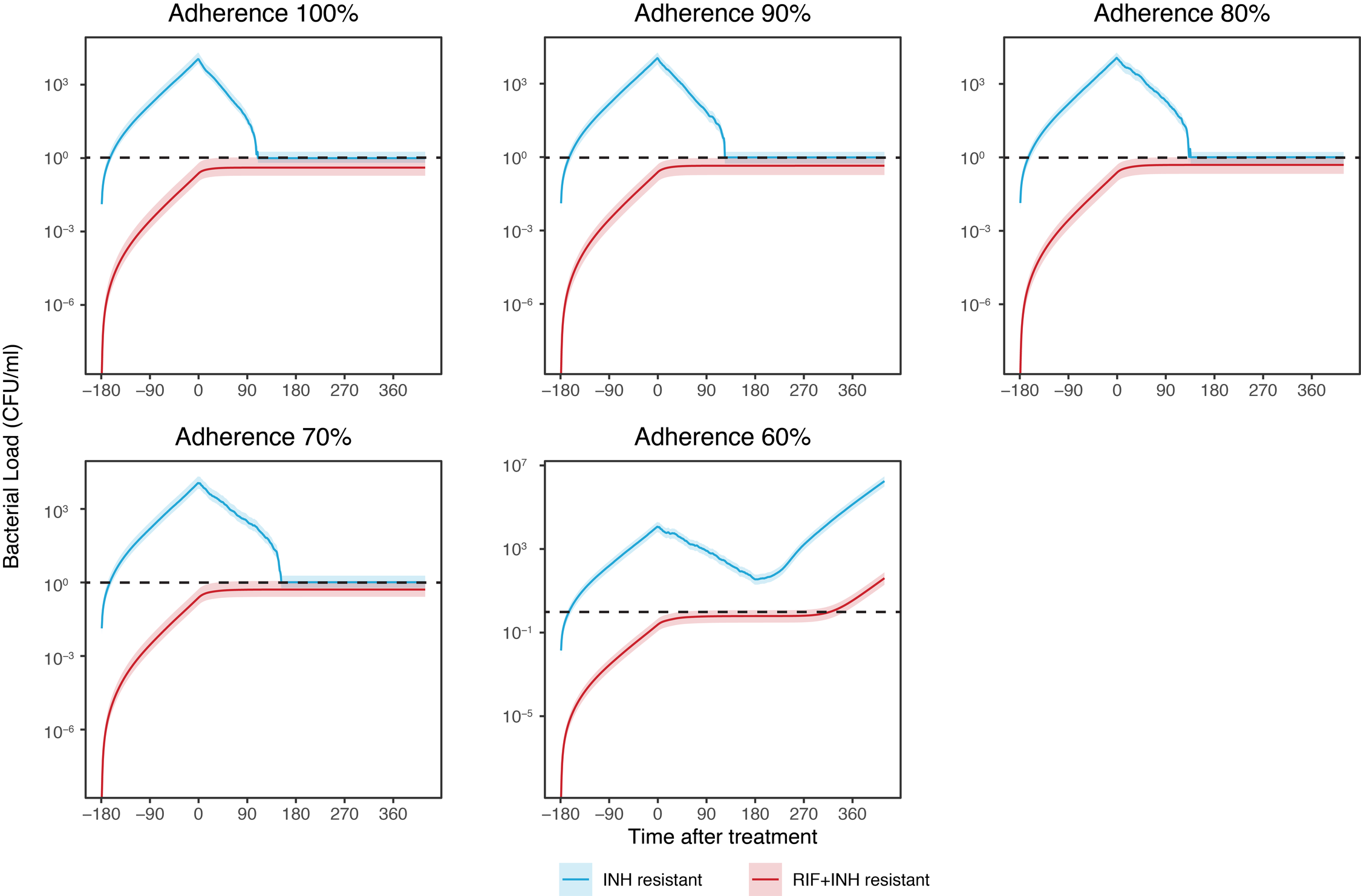

Supplement: S5 Fig — (TIF) [file pcbi.1008107.s005.tif]

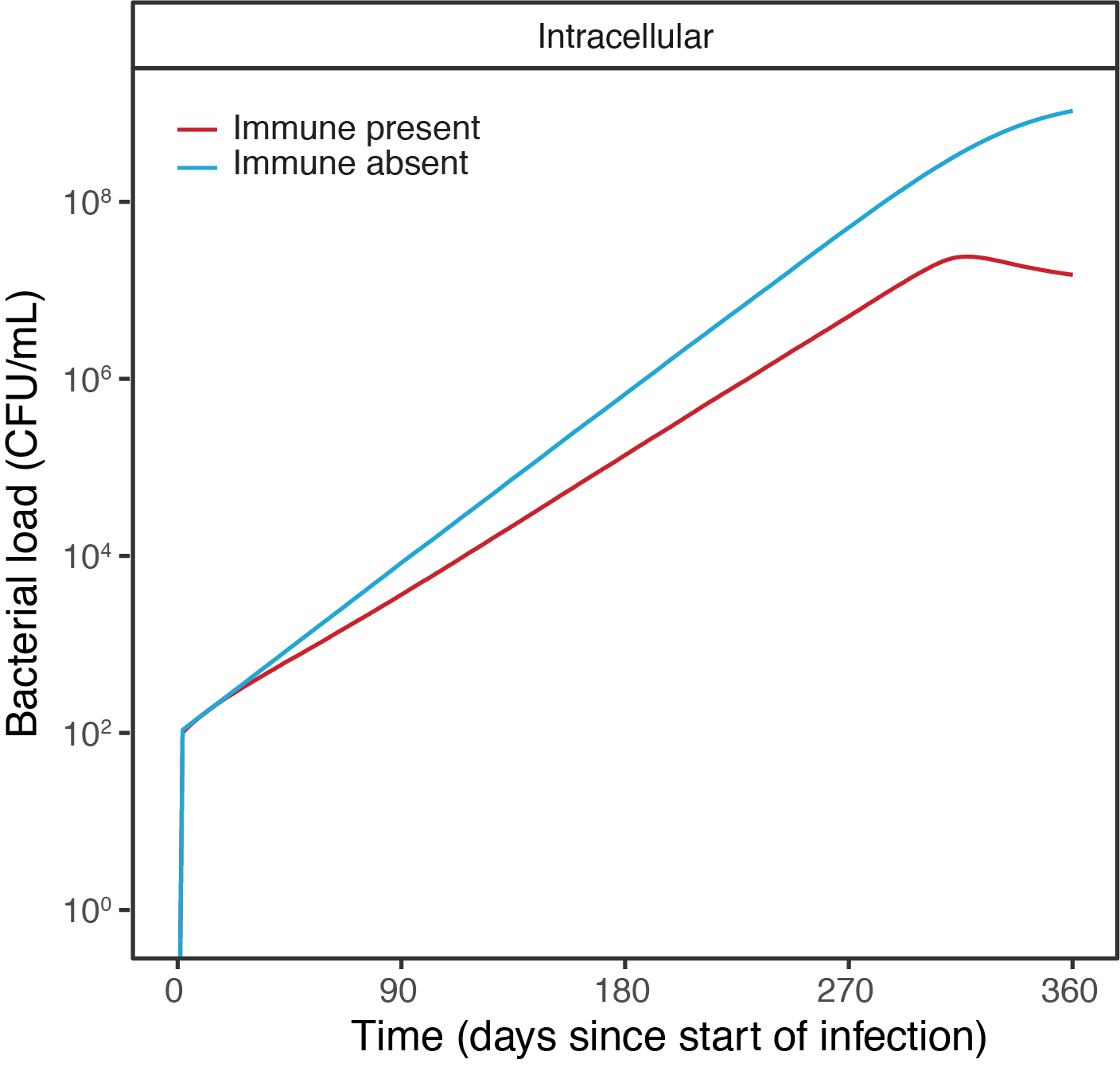

Supplement: S6 Fig — (TIF) [file pcbi.1008107.s006.tif]
