# Supplementary material for: Mathematical model and tool to explore shorter multi-drug therapy options for active pulmonary tuberculosis
Source: PLoS Comput Biol. 2020 Aug 18;16(8):e1008107. doi: 10.1371/journal.pcbi.1008107 (PMC7480878; doi:10.1371/journal.pcbi.1008107)
Supplement: S1 Text — (PDF) [file pcbi.1008107.s007.pdf]

Supporting Text 1 – Immune System Components

Immune System Dynamics

Immune system dynamics was represented by equations (A1) to (A17), with

- Lung components: macrophages (M_R, M_A, M_I), dendritic cells (IDC, MDC), T-cells (T_p, T_1, T_2), bacteria (B_i, B_e), cytokines ($IL_4, IL_{10}, IL_{12}, IFN_\gamma$)
- Lymph node components: cytokines ($IL12^{LN}$), T-cells (T^{LN}, T_p^{LN})

and using variable naming and parameter defintions as described in [76].

Macrophages in lung

$$\frac{dM_R}{dt} = S_M + \alpha_4(M_A + wM_I) - k_2 M_R\left(\frac{B_E}{B_E + c_9}\right) + k_4 M_A\left(\frac{IL_{10}}{IL_{10} + sc_8}\right)$$

$$-k_3 M_R\left(\frac{IFN_\gamma}{IFN_\gamma + f_3 IL_4 + sc_3}\right)\left(\frac{B_T}{B_T + c_8}\right) - \mu_R M_R \quad \text{(A1)}$$

$$\frac{dM_A}{dt} = k_3 M_R\left(\frac{IFN_\gamma}{IFN_\gamma + f_3 IL_4 + sc_3}\right)\left(\frac{B_T}{B_T + c_8}\right) - k_4 M_A\left(\frac{IL_{10}}{IL_{10} + sc_8}\right) - \mu_A M_A \quad \text{(A2)}$$

$$\frac{dM_I}{dt} = k_2 M_R\left(\frac{B_E}{B_E + c_9}\right) - k_{17} M_I\left(\frac{B_I^m}{B_I^m + (NM_I)^m}\right) - k_{14} M_I\left(\frac{T_1/M_I}{T_1/M_I + c_4}\right) - \mu_I M_I \quad \text{(A3)}$$

Cytokines in lung

$$\frac{dIL_4}{dt} = \alpha_{11} T_p + \alpha_{12} T_2 - \mu_{IL_4} IL_4 \quad \text{(A4)}$$

$$\frac{dIL_{10}}{dt} = \alpha_{14} M_A\left(\frac{sc_6}{IL_{10} + f_6 IFN_\gamma + sc_6}\right) + \alpha_{16} T_1 + \alpha_{17} T_2 + \alpha_{18} T_p + \delta_7 M_I - \mu_{IL_{10}} IL_{10} \quad \text{(A5)}$$

$$\frac{dIL_{12}}{dt} = \alpha_8 M_A + \alpha_{23} M_R - \mu_{IL_{12}} IL_{12} \quad \text{(A6)}$$

$$\frac{dIFN_\gamma}{dt} = s_g\left(\frac{B_T}{B_T + c_{10}}\right)\left(\frac{IL_{12}}{IL_{12} + sc_4}\right) + \alpha_5 T_1\left(\frac{M_A}{M_A + c_5}\right) - \mu_\gamma IFN_\gamma \quad \text{(A7)}$$

T-cells in lung

$$\frac{dT_p}{dt} = \xi T_p^{LN}\left(\frac{M_A}{M_A+\delta_6}\right) - k_6 IL_{12} T_p\left(\frac{IL_{12}^{LN}}{IL_{12}^{LN} + f_1 IL_4 + f_7 IL_{10} + sc_1}\right)$$

$$+\alpha_2 T_p\left(\frac{M_A}{M_A+c_{15}}\right) - k_7 T_p\left(\frac{IL_4}{IL_4 + f_2 IFN_\gamma + sc_2}\right) - \mu_{T_p} T_p \qquad \text{(A8)}$$

$$\frac{dT_1}{dt} = k_6 IL_{12} T_p\left(\frac{IL_{12}^{LN}}{IL_{12}^{LN} + f_1 IL_4 + f_7 IL_{10} + sc_1}\right) - \mu_{T_1} T_1 \qquad \text{(A9)}$$

$$\frac{dT_2}{dt} = k_7 T_p\left(\frac{IL_4}{IL_4 + f_2 IFN_\gamma + sc_2}\right) - \mu_{T_2} T_2 \qquad \text{(A10)}$$

Cytokines and T-cells in lymph node

$$\frac{dIL_{12}^{LN}}{dt} = \delta_1 MDC - \mu_{IL_{12}^{LN}} IL_{12}^{LN} \qquad \text{(A11)}$$

$$\frac{dT^{LN}}{dt} = S_{T^{LN}} + \delta_2 MDC - \lambda_1 T^{LN} - \mu_{T^{LN}} T^{LN} - \delta_4 T^{LN} * MDC \qquad \text{(A12)}$$

$$\frac{dT_p^{LN}}{dt} = \delta_4 T^{LN} * MDC + \delta_5 T_p^{LN}\left(1 - \frac{T_p^{LN}}{\rho}\right) - \xi T_p^{LN} \qquad \text{(A13)}$$

Dendritic cells

$$\frac{dMDC}{dt} = \varphi \delta_{10} IDC\left(\frac{B_E}{B_E+\delta_{11}}\right) - \mu_{MDC} MDC \qquad \text{(A14)}$$

$$\frac{dIDC}{dt} = S_{IDC} + \delta_8 IDC\left(\frac{B_E}{B_E+\delta_9}\right) - \delta_{10} IDC\left(\frac{B_E}{B_E+\delta_{11}}\right) - \mu_{IDC} IDC \qquad \text{(A15)}$$

Bacterial killing outside macrophages

$$\Delta B_E^{im} = -k_{15} M_A B_E - k_{18} M_R B_E + k_{14} N_1 M_I\left(\frac{T_1/M_I}{T_1/M_I + c_4}\right)$$

$$+ k_{17} N M_I\left(\frac{B_I^m}{B_I^m + (NM_I)^m}\right) - k_2\left(\frac{N}{2}\right) M_R\left(\frac{B_E}{B_E+c_9}\right) - \delta_{12} B_E IDC \qquad \text{(A16)}$$

Bacterial killing inside macrophages

$$\Delta B_I^{im} = -k_{17} N M_I \left(\frac{B_I^m}{B_I^m + (N M_I)^m} \right) + k_2 \left(\frac{N}{2} \right) M_R \left(\frac{B_E}{B_E + c_9} \right) - k_{14} N_1 M_I \left(\frac{T_1 / M_I}{T_1 / M_I + c_4} \right) \quad \text{(A17)}$$

Immune system effects were assumed to act equally on wild-type and different mono-resistant bacteria strains. Typical values for the immune system model parameters were based on data provided [76] except as described in Table T1. Patient-specific parameter values were generated using inter-individual variance, as well as stochastic errors applied at each time step. Initial values for the differential equation variables for TB infection are summarized in Table T2 below.

Table T1. Immune system model parameter values

Model parameter*	Value	Unit
Maximal T-cell killing rate (k_{14})	1.6	1/day
Rate of Th1 differentiation (k_6)	$1 \cdot 10^{-5}$	mL/pg/day
Rate of Th2 differentiation (k_7)	0.7	1/day
Extracellular bacterial growth rate (a_{20})	0.05	h^{-1}
Intracellular bacterial growth rate (a_{19})	0.09	h^{-1}
Bacterial uptake by IDC in lung (d_{12})	$1 \cdot 10^{-5}$	DC/mL/day
Half-sat, Be on IDC activation (d_{11})	100	Be/mL

* Note: all other immune model parameter values set per Marino & Kirschner (2004).

Table T2. Immune system model starting condition values

Parameter	Value	Unit
Resting macrophages	$5 \cdot 10^5$	cells/mL
Extracellular bacteria	100	CFU/mL
T cells	$4 \cdot 10^3$	cells/cm^3 of tissue
Immature dendritic cells	$5 \cdot 10^4$	cells/cm^3 of tissue

Note: parameter values reflect TB infection
