# Supplementary material for: Mathematical model and tool to explore shorter multi-drug therapy options for active pulmonary tuberculosis
Source: PLoS Comput Biol. 2020 Aug 18;16(8):e1008107. doi: 10.1371/journal.pcbi.1008107 (PMC7480878; doi:10.1371/journal.pcbi.1008107)
Supplement: S2 Text — (PDF) [file pcbi.1008107.s008.pdf]

Supporting Text 2 - Clinical Trial Data

Table 9. Clinical studies included in comparison of therapy vs. simulation outcome

Study	Lead Author	Year	N	Therapy Description*	Time (wks)	Dose	Est. Relapse	Menzies Relapse	Wallis Relapse	Wallis 2-mo CP	Simulation (%)
USA (HIV)	El Sadr [F]	1998	17 [8]	2wk H300R600Z2000E1200<br>6wk H900R600Z2500E1800 (3/wk)<br>18wk H900R600 (2/wk)	26	3/wk<br>2/wk	-	1/17<br>5.9%	-	-	11.7-15.3<br>CPCRA 6m
USA (HIV)	El Sadr et al.	1998	15 [8]	2wk H300R600Z2000E1200<br>6wk H900R600Z2500E1800 (3/wk)<br>31wk H900R600 (2/wk)	39	3/wk<br>2/wk	-	1/15<br>7.1%	-	-	6.7 - 9.7<br>CPCRA 9m
Iran	Sharifi-Mood [H]	2006	64 [1]	8wk H300R600Z1800E1200<br>16wk H300R600<br>(patient weight 60 kg)	24	Daily	-	6/64<br>9.4%	-	-	5.3 – 8.0<br>Shari_6m

Iran	Sharifi-<br>Mood [H]	200<br>6	33	8wk H300R600Z1800E1200<br>8wk H300R600<br>(patient weight 60 kg)	16	Daily	-	3/33<br>9.1%	-	-	19.3-23.7<br>Shari_4m
USPHS<br>Trial 21	Combs<br>[E]	199<br>0	271	8wk H300R600Z1800E900 (note<br>3)<br>16wk H300R600	24	Daily	13/284<br>[2]	10/385<br>2.6%	22/617<br>3.5%	0.18	5.0 – 7.7<br>Comb_6m
USPHS<br>Trial 21	Combs<br>[E]	199<br>0	212	8wk H300R600Z1800E900 (note<br>3)<br>31wk H300R600	39	Daily	7/219<br>[2]	6/231<br>2.6%	12/445<br>2.8%	0.27	0.3 – 1.3<br>Comb_9m
Zaire<br>(no HIV)	Perriens<br>[G]	199<br>5	144<br>[4]	8wk H300R720Z1800E1200<br>16wk H900R600 (2/wk)<br>(patient weight 60 kg)	24	2/wk	9/144<br>6.3%	-	-	-	5.0 - 8.0<br>Perrie 6m
Zaire<br>(with HIV)	Perriens<br>[G]	199<br>5	68<br>[4]	8wk H300R720Z1800E1200<br>16wk H900R600 (2/wk)	24	2/wk	-	9/68<br>13.2%	-	-	5.0 - 8.0<br>Perrie 6m

				(patient weight 60 kg)							
Zaire<br>(HIV)	Perriens<br>[G]	199<br>5	54<br>[4]	8wk H300R720Z1800E1200<br>44wk H900R600 (2/wk)<br>(patient weight 60 kg)	52	2/wk	-	1/54<br>1.9%	-	-	1.3 – 3.3<br>Perrie<br>12m
RIFAQUI<br>N	Jindani<br>[B]	201<br>4	167<br>[6]	8wk H300R600Z1500E1200<br>(note 7)<br>16wk H300R600<br>(patient weight 60 kg)	24	Daily	6/167<br>3.6%	-	-	-	3.3 – 5.2<br>TB4_6mo
REMox	Gillespie<br>[C]	201<br>4	498<br>[5]	8wk H300R600Z1500E1200<br>16wk H300R600<br>(patient weight 60 kg)	24	Daily	31/498<br>6.2%	-	-	-	3.3 – 5.2<br>TB4_6mo
Algeria	Algeria<br>[K]	199<br>1	518	8wk H300R600Z2000 (note 9)<br>20wk H300R600<br>(patient weight 60 kg)	28	Daily	-	23/618<br>4.7%	-	-	3.0 - 5.3<br>Algeria<br>6m

Algeria	Algeria [K]	1991	564	8wk H300R600Z2000 (note 9)<br>8wk H300R600<br>20wk H300 (patient weight 60 kg)	16 (RIF timing)	Daily	-	38/564 6.7%	-	-	3.0 - 5.0 Algeria 4m
Hong Kong	HK Chest Service	1987	160	24wk EHRZ (3/wk)	24	3/wk	-	-	3/160 1.9%	0.10	
Hong Kong	HK Chest Service	1987	163	24wk EHRZ (daily)	24	Daily	-	-	2/163 1.2%	0.06	
Singapore	Singapore	1988	109	2HRZ/4HR (3/wk)	24	3/wk	-	-	1/109 0.9%	0.10	
Global	Jindani	2004	347	2RHEZ/4HR	24	?	-	-	17/347 5.0%	0.17	
Uganda (HIV)	Okwera	2006	266	2HREZ/6HR	34	?	-	-	13/266 4.8%	0.02	

Uganda<br>(HIV)	Okwera	200<br>6	147	2HREZ/4HR	24	?	-	-	14/147<br>9.5%	0.05
Hong Kong	HK<br>BMRC	199<br>1	49	6SHRZ (3/wk)	24	3/wk	-	12/56<br>21.4%	10/49<br>20.0%	0.20
Hong Kong	HK<br>BMRC	199<br>1	50	8SHRZ (3/wk)	34	3/wk	-	2/42<br>4.8%	1/50<br>2.0%	0.20
Singapore	S BMRC	198<br>6	77	2SHRZ/2HR	16	Daily	-	7/87<br>8.1%	6/77<br>7.8%	0.02
Singapore	S BMRC	198<br>6	79	2SHRZ/2HRZ	16	Daily	-	10/89<br>11.2%	9/79<br>11.0%	0.02
Singapore	S BMRC	198<br>6	80	2SHRZ/4HR	24	Daily	-	2/85<br>2.4%	2/80<br>2.5%	0.02

Singapore	S BMRC	198<br>6	78	2SHRZ/4HRZ	24	Daily	-	0/82<br>0.0%	0/78<br>0.0%	0.02

* E = ethambutol, H = isoniazid, R = rifampin, Z = pyrazinamide

Note 1: excludes 3 patients with treatment failure

Note 2: includes both confirmed and suspected relapses

Note 3: only some patient received therapy including ethambutol

Note 4: excludes patients deceased or lost to follow up

Note 5: excludes patients with treatment failure and non-TB deaths

Note 6: excludes patients lost to follow up, inadequate treatment, and non-TB deaths

Note 7: excludes pyridoxine 25mg

Note 8: includes patients lost to follow up, and death to non-TB

Note 9: excluding streptomycin

Clinical Study References

[A] Menzies, D., Benedetti, A., Paydar, A., Martin, I., Royce, S., et al. (2009). Effect of duration and intermittency of rifampin on tuberculosis treatment outcomes: a systematic review and meta-analysis. *PLoS Med* 6(9), e1000146.

[B] Jindani, A., et al. (2014). High-dose rifapentine with moxifloxacin for pulmonary tuberculosis. *N Engl J Med.* 371(17), 1599-608.

[C] Gillespie, S., et al. (2014). Four-month moxifloxacin-based regimens for drug-sensitive tuberculosis. *N Engl J Med.* 371, 1577-1587.

[D] Felten, M. (1989). Importance of rifampicin in combined daily/intermittent chemotherapy for tuberculosis. *S Afr Med J.,* 75(11), 524-6.

[E] Combs, D., O'Brien, R., & Geiter, L. (1990). USPHS tuberculosis short-course chemotherapy trial 21: effectiveness, toxicity, and acceptability. *Ann Intern Med,* 112, 397-406.

[F] El-Sadr, W., Perlman, D., Matts, J., Nelson, E., Cohn, D., et al. (1998). Evaluation of an intensive intermittent-induction regimen and duration of short-course treatment for human immunodeficiency virus-related pulmonary tuberculosis. *Clinical Infectious Diseases,* 26, 1148-1158.

[G] Perriens, J., St.Louis, M., Mukadi, Y., Brown, C., Prignot, J., et al. (1995). Pulmonary tuberculosis in HIV-infected patients in Zaire. A controlled trial of treatment for either 6 or 12 months. *N Engl J Med* 332, 779-784.

[H] Sharifi-Mood, B., Metanat, M., Alavi-Naini, R., Kouhpayeh, H., Salehi, M., et al. (2006).The comparison of six-month and four-month regimens of chemotherapy in the treatment of smear positive pulmonary tuberculosis. *J Med Sci*, 6, 108-111.

[J] A Singapore-British Medical Research Council study (1978). Controlled trial of 4-month and 6-month regimens of chemotherapy in the treatment of pulmonary tuberculosis. *Ann Acad* Med 5, 242–243.

[K] Algerian Working Group British Medical Research Council Cooperative Study (1991). Short-course chemotherapy for pulmonary tuberculosis under routine programme conditions: a comparison of regimens of 28 and 36 weeks duration in Algeria. *Tuberc* 72, 88–100.

[L] Hong Kong Chest Service-Tuberculosis Research Centre MBMRC (1991) A controlled clinical comparison of 6 and 8 months of antituberculosis chemotherapy in the treatment of patients with silico tuberculosis in Hong Kong. *Am Rev Respir Dis* 143, 262–267.

[M] British Thoracic and Tuberculosis Association (1975) Short-course chemotherapy in pulmonary tuberculosis. *Lancet* 305: 119–124.

[N] British Thoracic and Tuberculosis Association, Angel H (1976) Short-course chemotherapy in pulmonary tuberculosis. a controlled trial by the British Thoracic and Tuberculosis Association. *Lancet* 2: 1102–1104.

[O] Singapore Tuberculosis Service-British Medical Research Council (1979). Clinical trial of six-month and four-month regimens of chemotherapy in the treatment of pulmonary tuberculosis. Am Rev Respir Dis 119: 579–585.

[P] Singapore Tuberculosis Service-British Medical Research Council (1981) Clinical trial of six-month and four-month regimens of chemotherapy in the treatment of pulmonary tuberculosis: the results up to 30 months. Tuberc 62: 95–102.

[Q] Singapore Tuberculosis Service-British Medical Research Council (1986) Long-term follow-up of a clinical trial of six-month and four-month regimens of chemotherapy in the treatment of pulmonary tuberculosis. Am Rev Respir Dis 133: 779–783.
