# Supplementary material for: Mathematical model and tool to explore shorter multi-drug therapy options for active pulmonary tuberculosis
Source: PLoS Comput Biol. 2020 Aug 18;16(8):e1008107. doi: 10.1371/journal.pcbi.1008107 (PMC7480878; doi:10.1371/journal.pcbi.1008107)
Supplement: S3 Text — (PDF) [file pcbi.1008107.s009.pdf]

Supporting Text 3: TB Drug Models and Parameters

Introduction

This document is a repository for information on models and parameters for pharmacokinetics / pharmacodynamics of commonly analyzed drugs. It describes the current models, their key assumptions and definitions, as well as relevant references and source information.

Individual drugs

- Isoniazid (INH, H)
- Rifampicin (RIF, R)
- Ethambutol (EMB, E)
- Pyrazinamide (PZA, Z)

Isoniazid

Introduction

Isoniazid is bactericidal for intra- and extracellular mycobacteria and exerts its action by inhibiting synthesis of mycolic acid needed for maintaining the mycobacterial cell wall. Isoniazid is a prodrug which becomes activated by a bacterial catalase-peroxidase enzyme in *M. tuberculosis*. Isoniazid is bactericidal to rapidly dividing mycobacteria, but is bacteriostatic if the mycobacteria are slow-growing. Some studies even suggest isoniazid has minimal effect on slow-growing bacteria.

Isoniazid inhibits the P450 system and thus may interact with several other medications metabolized in liver, such as phenytoin and warfarin. Isoniazid is metabolized in the liver via acetylation. Two forms of the enzyme are responsible for acetylation, so some patients metabolize the drug more quickly than others. The protein binding of isoniazid is estimated at only 1 to 10%, and therefore protein binding in ELF and CSF generally is negligible.

Isoniazid - Pharmacokinetic model and parameters

The PK model is based on a single-compartment system with first-order absorption and elimination. Some models include a multi-step portion for absorption phase. Transfer from extracellular to intracellular (macrophage) space is based on IO ratio, and assumed to occur rapidly, with relatively minimal extended bacterial effect. Diffusion into granuloma is assumed to occur based GR ratio. Elimination rate depends on if individual is assigned as slow or fast acetylator, based on ratio Ae.

Param	Description	UOM	Value	CV	Source	Population & comments
Ka	Coefficient of absorption (oral)	1/h	1.85<br>2.57<br>**2.21**	0.90<br>0.62<br>**0.76**	[H3] Table 2<br>[H4] Table 2	SA TB patients (n=235)<br>Healthy volunteers (n=24)
CL/F	Total clearance (single dose)	L/h	19.8<br>25.8<br>9.7<br>15.9<br>**17.8**	-<br>-<br>-<br>0.20<br>**0.20**	[H1] Table 2<br>[H2] Table 2<br>[H3] Table 2<br>[H4] Table 3	Dutch TB patients (n=12)<br>Tanzania TB patients (n=19)<br>Value for slow acetylators<br>Value for slow acetylators
V/F	Effective volume	L	82<br>99<br>58<br>62<br>**75**	-<br>-<br>0.16<br>0.17<br>**0.17**	[H1] Table 2<br>[H2] Table 2<br>[H3] Table 3<br>[H4] Table 2	Dutch TB patients (n=12)<br>Tanzania TB patients (n=19)<br>SA TB patients (n=235)<br>Scaled to body mass 65 kg
Ke (CL/V)	Coefficient of elimination (total)	1/h	(0.24)<br>(0.26)<br>0.17<br>0.26<br>**0.22**	-<br>-<br>0.18<br>0.18<br>**0.18**	[H1]<br>[H2]<br>[H3]<br>[H4]	Slow acetylators<br>Slow acetylators<br>Based on 0.17 and 0.26

Ae mult	Fast vs. slow acetylation rate effect on clearance	-	2.22<br>2.77<br>2.54<br>2.47<br>**2.50**	-<br>-<br>-<br>-	[H3] Table 2<br>[H4] Table 1<br>[H19] Table 4<br>[H20] Table 5	Based on CL/F 21.6 / 9.7<br>Based on Cmax 0.61 / 0.22<br>Based on CL/F 11.3 / 4.44<br>Multiple studies, pediatric
Ae ratio	Share of fast vs. slow acetylators (expressed as fast share of total pop.)	-	0.13<br>0.33<br>0.61<br>0.69<br>0.49<br>**N/A***	-	[H3] Table 2<br>[H4] p 2672<br>[H19] p 1344	SA TB patients (n=235)<br>For particular study sample<br>Fast and intermediate<br>Polish TB study (n=237)<br>Pakistan study (n=54)
IO factor	Intracellular ratio (inside vs. outside macrophage)	-	0.9<br>1.0<br>**0.95****	-	[H6] p 933<br>[H21] p 2363	Healthy volunteers, old data<br>Adult volunteers (n=80)<br>Based on 0.9 and 1.0
GR factor	Granuloma ratio (inside vs. outside granuloma)***	-	0.28	-	[H5] Fig 8	Rabbit lesions (n=25).<br>Based on 0.07 / 0.25

* Proportion of fast vs. slow acetylators based on any specific target population. For most simulations a value of 0.50 is used.

** Concentration in lung tissue found to be far less than in plasma (Kjellsson et al. 2012). On the other hand, concentration of drug inside the bacteria is believed to be significantly higher than concentration outside bacteria, and it was estimated at 4-5 times by Bardou et al. (1998) [H23]. The net effect of these factors may be a concentration (intrabacterial vs. plasma) of approx 1.

*** Granuloma concentration ratio is believed to be heterogeneous, depending on factors such as size and caseation - which influence hypoxic state and drug diffusion characteristics.

Isoniazid - Pharmacodynamics

Individual drugs assumed to act independently with a) bactericidal *Bk*, and b) bacteriostatic effect *Bg*, where
Bk = 1/(1+(EC50k/C)^ak)) * k(t), for time t, in a given model compartment, *added* for N drugs
Bg = (1-1 / (1 + (EC50g/C)^ag)), *multiplied* for N drugs

Kill rate for isoniazid is believed to be highest for fast-growing bacteria, and it is far less effective on persisting bacteria, thus k(t) is set to 25% of kmax when t >15 days, as detailed further below.

Param	**Description**	**UOM**	**Value**	**CV**	**Source**	**Population & comments**
EC50k	EC50 for bacterial killing	mg/L	(4.42)<br>(0.244)<br>0.36<br>**0.40**	-<br>2.71<br>-<br>-	[H8] Fig 1<br>[H24] Table 2<br>[H25] IC50	Possibly some resistance effects<br>HFS, using *M. bovis*<br>Santa Cruz Biotech<br>Selected value
EC50g	EC50 for growth inhibition	mg/L	(3.24)<br>0.1	0.41<br>-	[H16] Table 1	HFS, non-resistant strain<br>Estimated as 2 x MIC
MIC	Minimal inhibitory concentration	mg/L	0.016<br>0.031<br>0.05<br>0.038<br>0.11<br>**0.05**	-<br>0.25<br>-<br>-<br>-<br>**0.25**	[H9] p 2330<br>[H11] Table 2<br>[H12]<br>[H17]<br>[H22] p 1322	HFS<br>in vitro, bioluminescence<br><br>“0.025 to 0.05”<br>“0.02 to 0.2”
MIC ratio	Intra- vs. extra-cellular MIC ratio	-	1	-		No source
α_k	Hill curve factor (bactericidal)	-	0.9<br>1.0<br>1.0<br>1.28<br>**1.05**	0.4<br>0.4<br>-<br>0.06<br>**0.29**	[H8] Table 3<br>[H8] Table 3<br>[H8] Table 3<br>[H16] Table 1	HFS<br>Human sputum<br>Mouse<br>HFS, non-resistant strain
α_g	Hill curve factor (bacteriostatic)	-	(3.8)<br>1.05	-	[H16] Table 1	HFS, non-resistant strain<br>Use same value as α_k
Emax extracell	Max kill rate (log10)	CFU/ ml/ day	4.4<br>(2.89)<br>4.0<br>3.8<br>(0.65)<br>(2.25)<br>**4.1**	-<br>-<br>-<br>-<br>-<br>-	[H7] Fig 1<br>[H9] p 2331<br>[H10] p 2955<br>[H15] Fig 2A<br>[H15] Fig 2B<br>[H16] Fig 3	In-vitro agar plates<br>HFS<br>In-vitro<br>In-vitro (exponential phase)<br>In-vitro (stationary phase)<br>HFS<br>Based on highest 3 values
Emax intracell	Max kill rate (log10)	CFU/ ml/ day	0.5<br><br>0.5	-	[H10] p 2955	In-vitro mouse macrophage<br>“Only bacteriostatic effect”

$kill_e$	Max kill rate per hour in base e	1/h	0.39	-		Transformed by ln(10)/24 **d0d2d15: 0.4>0.2>0.1***
$kill_i$	Max kill rate per hour in base e	1/h	0.05	-	Likely a very small effect	Transformed by ln(10)/24 **d0d2d15: 0.05>0.02>0.01***
mutation rate	Mutation rate for mono-resistance	1/gen	2.6e-8<br>2.5e-8<br>1.6e-8<br>**2.2e-8**	-<br>-<br>-	[H12] p 269<br>[H13] p S26<br>[H14] p 515	In-vitro plates & macrophages<br>From: 3.2×10−7* 5% (est)
mutation rate 1/h	Mutation rate for mono-resistance	1/h	**9.2e-10**	-		Divide per-generation rate by 24 hr (est. growth cycle)

* Kill rate reduces quickly for slower growing bacteria, see [H26] Table 7

Isoniazid - References

[H1] Magis-Escurra, C., et al. (2014). Population pharmacokinetics and limited sampling strategy for first-line tuberculosis drugs and moxifloxacin. *Int J Antimicrob Agents.* pii: S0924-8579(14)00164-2. PMID: 24985091

[H2] Tostmann, A., et al. (2013) Pharmacokinetics of first-line tuberculosis drugs in Tanzanian patients. *Antimicrob Agents Chemotherapy.* 57(7), 3208-3213. PMID: 23629715

[H3] Wilkins, J., et al. (2011). Variability in the population pharmacokinetics of isoniazid in South African tuberculosis patients. *Br J Clin Pharmacol.* 72(1), 51-62. PMID: 23629715

[H4] Peloquin CA, et al. 1997. Population pharmacokinetic modeling of isoniazid, rifampin, and pyrazinamide. *Antimicrob. Agents Chemother.* 41, 2670-2679. PMID: 9420037

[H5] Kjellsson, M., et al. (2012). Pharmacokinetic evaluation of the penetration of antituberculosis agents in rabbit pulmonary lesions. *Antimicrobial Agents and Chemotherapy*, 56(1), 446–457. PMID: 21986820

[H6] Hand, W., et al. (1984). Uptake of antibiotics by human alveolar macrophages. *Am Rev Respir Dis.* 129(6):933-7. PMID: 6732052

[H7] Steenwinkel, J., et al. (2010). Time-kill kinetics of anti-tuberculosis drugs, and emergence of resistance, in relation to metabolic activity of *Mycobacterium tuberculosis*. *J Antimicrob Chemother.* 65(12), 2582-2589. PMID: 20947621

[H8] Pasipanodya JG, et al. (2011). An oracle: antituberculosis pharmacokinetics-pharmacodynamics, clinical correlation, and clinical trial simulations to predict the future. *Antimicrob. Agents Chemother.* 55, 24–34. PMID: 2093777

[H9] Gumbo, T. et al. (2007). Isoniazid bactericidal activity and resistance emergence: integrating pharmacodynamics and pharmacogenomics to predict efficacy in different ethnic populations. *Antimicrob Agents Chemother.* 51(7), 2329-2336. PMID: 17438043

[H10] Jayaram, R., et al. (2004). Isoniazid pharmacokinetics-pharmacodynamics in an aerosol infection model of tuberculosis. *Antimicrob. Agents Chemother.* 48(8), 2951–2957. PMID: 15273105

[H11] Andreu, N. et al. (2007). Rapid measurement of antituberculosis drug activity in vitro and in macrophages using bioluminescence. *J Antimicrob Chemother.* 67(2), 404–414. PMID: 22101217
[H12] Gillespie, S. (2002). Evolution of drug resistance in *Mycobacterium tuberculosis*: clinical and molecular perspective. *Antimicrob. Agents Chemother.* 46(2), 267-274. PMID: 11796329
[H13] Nachega, J. & Chaisson, R. (2003). Tuberculosis drug resistance: a global threat. *Clinical Infectious Diseases*, 36(Suppl 1), S24–30. PMID: 12516027
[H14] Bergval, I. et al. (2009). Resistant mutants of *Mycobacterium tuberculosis* selected *in vitro* do not reflect the *in vivo* mechanism of isoniazid resistance. *J. Antimicrob. Chemother*. 64 (3), 515-523. PMID: 19578178
[H15] Paramasivan, S. et al. (2005). Bactericidal action of gatifloxacin, rifampin, and isoniazid on logarithmic- and stationary-phase cultures of *Mycobacterium tuberculosis*. *Antimicrob Agents Chemother.* 49(2), 627–631. PMID: 15673743
[H16] Gumbo, T. et al. (2007). Isoniazid's bactericidal activity ceases because of the emergence of resistance, not depletion of *Mycobacterium tuberculosis* in the log phase of growth. *J Infect Dis.* 195(2), 194-201. PMID: 17191164
[H17] Heifets, L. B. (1991). Antituberculosis drugs: anti-microbial activity in vitro, p. 13–58. In L. B. Heifets (ed.), *Drug susceptibility in the chemotherapy of mycobacterial infections*. Boca Raton, Fla.: CRC Press. ISBN-13: 978-0849367168
[H18] Dartois, V. (2014). The path of anti-tuberculosis drugs: from blood to lesions to mycobacterial cells. *Nature Reviews Microbiology.*12, 159–167. PMID: 24487820
[H19] Zvada, S., et al. (2014). Population pharmacokinetics of rifampicin, pyrazinamide and isoniazid in children with tuberculosis: in silico evaluation of currently recommended doses. *J Antimicrob Chemother.* 69(5), 1339-1349. PMID: 24486870
[H20] Ramachandran,G., et al. (2011). Pharmacokinetics of anti-tuberculosis drugs in children. *The Indian Journal of Pediatrics.* 78(4), 435-442. PMID: 21165722
[H21] Conte, J. Jr, et al. (2002).Effects of gender, AIDS, and acetylator status on intrapulmonary concentrations of isoniazid. *Antimicrob Agents Chemother.* 46(8), 2358-2364. PMID: 12121905
[H22] Zhang Y., & Yew, W. (2009). Mechanisms of drug resistance in *Mycobacterium tuberculosis. Int J Tuberc Lung Dis.* 13(11), 1320-1330. PMID: 19861002
[H23] Bardou, F. et al. (1998). Mechanism of isoniazid uptake in *Mycobacterium tuberculosis*. *Microbiology*. 144(Pt 9), 2539-2544. PMID: 9782502
[H24] Budha, N. et al. (2009). A simple in vitro PK/PD model system to determine time-kill curves of drugs against mycobacteria. *Tuberculosis* 89(5), 378–385. PMID: 19748318
[H25] Santa Cruz Biotech (2014). Isoniazid (CAS 54-85-3) accessed on Aug 28 from http://www.scbt.com/datasheet-205722-isoniazid.html
[R26] Mitchison, D. & Davies, G.(2008). Assessment of the efficacy of new anti-tuberculosis drugs. *Open Infect Dis J.* 2: 59–76. PMID: 23814629

Rifampicin (Rifampin)

Introduction

Rifampicin is bactericidal for intra- and extracellular bacteria. Rifampicin inhibits bacterial DNA-dependent RNA synthesis by inhibiting bacterial DNA-dependent RNA polymerase. Rifampicin may be bacteriostatic or bactericidal depending on the concentration of drug attained at site of infection. The bactericidal actions are secondary to interfering with the synthesis of nucleic acids by inhibiting bacterial DNA dependent RNA polymers at the B-subunit thus preventing initiation of RNA transcription, but not chain elongation.

Rifampicin will bind to human plasma proteins at around 90% among healthy individuals, and potentially slightly less (approx. 86%) among TB patients (Boman & Ringberger, 1974). Plasma binding is believed to not be significantly influenced by concurrent administration of other common TB drugs. The half-life of rifampicin is typically around 2.5 hours, with a range from 1.5 to 5.0 hours, though hepatic impairment will significantly increase it. Rifampicin is an inducer of many enzymes of the cytochrome P450 superfamily, and also results in significant autoinduction. The oral bioavailability is reduced by food and by first-pass metabolism. When rifampicin is taken with a meal, peak blood concentration falls by about a third.

Rifampicin - Pharmacokinetic model and parameters

The PK model is based on a single-compartment system with first-order absorption and elimination. Some models include a multi-step portion for absorption phase. Transfer from extracellular to intracellular (macrophage) space is based on IO ratio, and assumed to occur rapidly, and have relatively minimal extended bacterial effect. Diffusion into granuloma is assumed to occur based GR ratio. Autoinduction effects are applied based on number of days of drug administration during preceding period.

Param	Description	UOM	Value	CV	Source	Population & comments
Ka	Coefficient of absorption (oral)	1/h	2.00<br>1.83<br>1.61<br>**1.81**	-<br>0.53<br>0.43<br>**0.48**	[R18] p 83<br>[R1] Table 7 (lag)<br>[R6] Table 2	Multiple underlying sources<br>Healthy volunteers (n=24)<br>SA TB patients (n=261)
CL/F	Total clearance (single dose)	L/h	8.5<br><br>8.67<br>14.4**<br>14.5**<br>19.2**<br>**8.1***	0.30<br><br>0.27<br>-<br>-<br>0.53<br>**0.37**	[R11] p 2095<br><br>[R1] Table 7 (lag)<br>[R2] Table 2<br>[R3] Table 2<br>[R6] Table 3	TB & HIV pts, Africa (n=174), scaled from 55 to 60 kg<br>Healthy volunteers (n=24)<br>Tanzania TB patients (n=20)<br>Dutch TB patients (n=33)<br>SA TB patients (n=261)<br>Last 3 values adjusted for auto-induction (x 0.5)
V/F	Effective volume	L	74.3<br>33.2<br>37.3**<br>39.2**	0.19<br>0.10<br>-<br>-	[R11] page 2095<br>[R1] Table 7 (lag)<br>[R2] Table 2<br>[R3] Table 2	Scaled from 70 to 60 kg<br>Healthy volunteers (n=24)<br>Tanzania TB patients (n=20)<br>Dutch TB patients (n=33)

			63.8**<br><br>**49.6**	0.43<br><br>**0.24**	[R6] Table 3	SA TB patients (n=261), scale from 50 to 60 kg
Ke (CL/V)	Coefficient of elimination (total)	1/h	0.11<br>0.26<br>0.39**<br>0.37**<br>0.30**<br>0.30**<br>**0.18***	0.30<br>0.27<br>-<br>-<br>-<br>0.46<br>**0.34**	[R11]<br>[R1]<br>[R2]<br>[R3]<br>[R6]<br>[R7]	Calculated based on CL/V<br><br><br><br><br>TB patients, HIV+ (n=31)<br>Last 4 values adjusted for auto induction (x 0.5)
Ke mult	Auto-induction multiplier	-	1.85<br>2.18<br>**2.02**	-<br>-	[R11] page 2091<br>[R6] TBD	TB & HIV pts, Africa (n=174)<br>see above
Ke time	Auto-induction time	days	20(e)<br>14(e)<br>**17**	-<br>-	[R11] page 2096<br>[R6] TBD	"50% effect in approx. 8 days"<br>50% effect in approx. 6 days
IO factor	Intracellular ratio (inside vs. outside macrophage)	-	3.5<br>2.0<br>4.4<br>5.0<br>**3.7**	-<br>-<br>0.43<br>-<br>**0.43**	[R5] p 455<br>[R8] p 3785<br>[R4] Table 3<br>[R15] Table 2	Mtb infection in rabbits (ELF)<br>Est. Intra- vs. extracellular<br>In vitro, human macrophages<br>Mouse cell macrophages
GR factor	Granuloma ratio (inside vs. outside granuloma)***	-	**0.11**	-	[R5] p 455	Mtb infection in rabbits. Based on 0.4 / 3.5 (GR/ELF)

* Excluding auto-induction effect
** Sampling done following a period of steady-state drug administration

*** Granuloma concentration ratio is believed to be heterogeneous, depending on factors such as size and caseation - which influence hypoxic state and drug diffusion characteristics.

Rifampicin - Pharmacodynamics

Individual drugs assumed to act independently with a) bactericidal *Bk*, and b) bacteriostatic effect *Bg*, where

Bk = 1/(1+(EC50k/C)^ak)) * k(t), for time t, in a given model compartment, added for N drugs

Bg = (1-1 / (1 + (EC50g/C)^ag)), multiplied across N drugs

Param	Description	UOM	Value	CV	Source	Comment
EC50k	EC50 for bacterial killing	mg/L	7.65<br>4.72<br>4.71<br>5.7<br>**5.0**	0.02<br>-<br>-<br>-<br>-	[R8] Table 2<br>[R19] Table 1<br>[R9]<br>[R20]	HFS, non-resistant strain<br>Mouse<br>TBD<br>Liquid culture<br>Value selected
EC50g	EC50 for growth inhibition	mg/L	1.93<br>**1.5**	0.30	[R8] Table 2	HFS, non-resistant strain<br>Value selected
MIC	Minimal inhibitory concentration	mg/L	0.2<br>0.25<br>0.1<br>**0.18**	-	[R21] Table 3<br>[R15]<br>[R9]	TBD<br>Mouse cell macrophages<br>In-vitro agar plates
MIC ratio	Intra- vs. extra-cellular MIC ratio	-	3	0.17	[R15] Table 2	Value is in range of 2-4x
αk	Hill curve factor (bactericidal)	-	1.39<br>0.7(ex)<br>0.48(in)<br>0.60<br>**0.79**	0.70<br>-<br>-<br>-<br>**0.70**	[R8] Table 2<br>[R9] p 2120<br>[R9] p 2121<br>[R19] Table 1	HFS, non-resistant strain<br>In-vitro agar plates<br>In-vitro mouse macrophages<br>Computation
αg	Hill curve factor (bacteriostatic)	-	**0.36**	0.33	[R8] Table 2	HFS, non-resistant strain
Emax extracell	Max kill rate (log10)	CFU/ ml/ day	6.0<br>5.8<br>(0.9)<br>(1.6)<br>**5.9**	-<br>-<br>-<br>-<br>-	[R9] Fig 1A<br>[R10] Fig 1<br>[R16] Fig 1A<br>[R16] Fig 3B	In-vitro agar plates<br>In-vitro agar plates<br>In-vitro, exp. phase (1mg/L)<br>In-vitro, stat. phase (1mg/L)<br>Based on first 2 values
Emax intracell	Max kill rate (log10)	CFU/ ml/d	(0.6)	-	[R9] Fig 3A	In-vitro mouse macrophages<br>Assume is same as extracell.
$kill_e$	Max kill rate per hour in base e	1/h	0.57	-		Transformed by ln(10)/24<br>**d0d2d15: 0.5>0.4>0.4***
$kill_i$	Max kill rate per hour in base e	1/h	same as $kill_e$	-		Transformed by ln(10)/24<br>**d0d2d15: 0.5>0.4>0.4***
mutation rate	Mutation rate for mono-resistance	1/gen	2.3e-8<br>3.3e-9<br>2.2e-10<br>**8.8e-9**	-<br>-<br>-<br>**-**	[R12] p 1289<br>[R13] p 269<br>[R14] p S24	In-vitro plates & macrophages

mutation rate 1/h	Mutation rate for mono-resistance	1/h	**3.7e-10**	-		Divide per-generation rate by 24 hr (est. growth cycle)

* Similar kill rate for growing and stationary bacteria, see [H26] Table 7

Rifampicin - References

[R1] Peloquin CA, et al. 1997. Population pharmacokinetic modeling of isoniazid, rifampin, and pyrazinamide. *Antimicrob. Agents Chemother.* 41, 2670-2679. PMID: 9420037

[R2] Tostmann, A., et al. (2013) Pharmacokinetics of first-line tuberculosis drugs in Tanzanian patients. *Antimicrob Agents Chemotherapy.* 57(7), 3208-3213. PMID: 23629715

[R3] Magis-Escurra, C., et al. (2014). Population pharmacokinetics and limited sampling strategy for first-line tuberculosis drugs and moxifloxacin. *Int J Antimicrob Agents.* pii: S0924-8579(14)00164-2. PMID: 24985091

[R4] Mor, N., et al. (1995). Comparison of activities of rifapentine and rifampin against *Mycobacterium tuberculosis* residing in human macrophages. *Antimicrob. Agents Chemother.* 39(9):2073-2077. PMID: 8540718

[R5] Kjelsson, M., et al. (2012). Pharmacokinetic evaluation of the penetration of antituberculosis agents in rabbit pulmonary lesions. *Antimicrobial Agents and Chemotherapy*, 56(1), 446-457. PMID: 21986820

[R6] Wilkins, J., et al. (2008). Population pharmacokinetics of rifampin in pulmonary tuberculosis patients, including a semimechanistic model to describe variable absorption. *Antimicrob Agents Chemother*. 52(6), 2138-2148. PMID: 18391026

[R7] Perlman, D. et al. (2005). The clinical pharmacokinetics of rifampin and ethambutol in HIV-infected persons with tuberculosis. *Clin Infect Dis*, 41(11), 1638-1647. PMID: 16267738

[R8] Gumbo, T., et al., (2007). Concentration-dependent M*ycobacterium tuberculosis* killing and prevention of resistance by rifampin. *Antimicrobial Agents and Chemotherapy,* 51(11), 3781-3788. PMID: 17724157

[R9] Jayaram et al. (2003). Pharmacokinetics-pharmacodynamics of rifampin in an aerosol infection model of tuberculosis. *Antimicrobial Agents and Chemoth*erapy, 47(7), 2118-2124. PMID: 12821456

[R10] Steenwinkel, J., et al. (2010). Time-kill kinetics of anti-tuberculosis drugs, and emergence of resistance, in relation to metabolic activity of *Mycobacterium tuberculosis*. *J Antimicrob Chemother.* 65(12), 2582-2589. PMID: 20947621

[R11] Smythe W, et al. (2012). A semimechanistic pharmacokinetic-enzyme turnover model for rifampin autoinduction in adult tuberculosis patients. *Antimicrob. Agents Chemother.* 56(4), 2091–2098. PMID: 22252827

[R12] Mariam, D. et al. (2004). Effect of rpoB mutations conferring rifampin resistance on fitness of *Mycobacterium tuberculosis*. *Antimicrobial Agents and Chemotherapy*, 48(4), 1289–1294. PMID: 15047531

[R13] Gillespie, S. (2002). Evolution of drug resistance in *Mycobacterium tuberculosis*: clinical and molecular perspective. *Antimicrob. Agents Chemother.* 46(2), 267-274. PMID: 11796329

[R14] Nachega, J. & Chaisson, R. (2003). Tuberculosis drug resistance: a global threat. *Clinical Infectious Diseases*, 36(Suppl 1), S24–30. PMID: 12516027

[R15] Burman, W. (1997). The value of in vitro drug activity and pharmacokinetics in predicting the effectiveness of antimycobacterial therapy: a critical review. *The American Journal of the Medical Sciences*. 313(6), 355-363. PMID: 9186151
[R16] Paramasivan, S. et al. (2005). Bactericidal action of gatifloxacin, rifampin, and isoniazid on logarithmic- and stationary-phase cultures of *Mycobacterium tuberculosis*. *Antimicrob Agents Chemother.* 49(2), 627–631. PMID: 15673743
[R17] Pasipanodya JG, et al. (2011). An oracle: antituberculosis pharmacokinetics-pharmacodynamics, clinical correlation, and clinical trial simulations to predict the future. *Antimicrob. Agents Chemother.* 55, 24–34. PMID: 2093777
[R18] Goutelle, S., et al. (2011). Mathematical modeling of pulmonary tuberculosis therapy: Insights from a prototype model with rifampin. *J Theor Biol.* 282(1), 80-92. PMID: 21605569
[R19] Lyons M. (2014). Computational pharmacology of rifampin in mice: an application to dose optimization with conflicting objectives in tuberculosis treatment. *J Pharmacokinet Pharmacodyn.* DOI: 10.1007/s10928-014-9380-2. PMID: 25173151
[R20] Steenwinkel, de, J., et al. (2012). Drug susceptibility of *Mycobacterium tuberculosis* Beijing genotype and association with MDR TB. *Emerg Infect Dis.* 18(4), 660–663. PMID: 22469099
[R21] Mitchison, D. & Davies, G.(2008). Assessment of the efficacy of new anti-tuberculosis drugs. *Open Infect Dis J.* 2: 59–76. PMID: 23814629
[R22] Xie, Z. et al. (2005). Differential antibiotic susceptibilities of starved *Mycobacterium tuberculosis* isolates. *Antimicrob Agents Chemother.* 49(11), 4778–4780. PMID: 16251329
[R23] N.A. (2008) Rifampin. *Tuberculosis* 88(2), 151–154. PMID: 18486058

Pyrazinamide

Introduction

Pyrazinamide is bactericidal in an acidic environment, such as for slow growing bacteria in macrophages and granuloma tissue. It is believed many TB patients have chronically inflamed lungs, which may contribute to a more acidic environment. The drug has a sterilizing effect and is believed to be most active against dormant slow growing (“persisting”) bacteria, thereby allowing the treatment period to be shortened. The activity focused on slow-growing bacteria is opposite that of many other drugs used in TB therapy, which act best on actively growing bacteria.

Pyrazinamide - Pharmacokinetic model and parameters

The PK model is based on a single-compartment system with first-order absorption and elimination. Transfer between extra- and intracellular (macrophage) space is based on IO ratio, and assumed to occur rapidly, and have relatively minimal extended bacterial effect. Diffusion into granuloma is assumed to occur based GR ratio. Auto-induction effects are assumed to be non-significant for Pyrazinamide.

Param	Description	UOM	Value	CV	Source	Population & comments
Ka	Coefficient of absorption (oral)	1/h	3.25<br>3.58<br>**3.42**	0.72<br>0.52<br>**0.62**	[Z4] Table 9<br>[Z6] page 3199	Healthy volunteers (n=24)
CL/F	Total clearance (single dose)	L/h	4.5<br>4.6<br>3.7<br>**4.3**	-<br>-<br>0.16<br>**0.16**	[Z1] Table 2<br>[Z2] Table 2<br>[Z4] Table 9	Tanzania TB patients (n=20)<br>Dutch TB patients (n=19)<br>Healthy volunteers (n=24)
V/F	Effective volume	L	40.3<br>37.0<br>40.8<br>**39.4**	-<br>-<br>0.09<br>**0.09**	[Z1] Table 2<br>[Z2] Table 2<br>[Z4] Table 9	Tanzania TB patients (n=20)<br>Dutch TB patients (n=19)<br>Scaled to 60 kg body mass
Ke (CL/V)	Coefficient of elimination (total)	1/h	0.112<br>0.124<br>0.091<br>0.09<br>**0.104**	-<br>-<br>0.16<br>0.30<br>**0.23**	[Z1]<br>[Z2]<br>[Z4]<br>[Z5]	Based on CL/V above<br><br><br>TB patients HIV+ (n=48)
Ke mult	Auto-induction multiplier	-	1.0			No auto induction
Ke time	Auto-induction time	days	-			No auto induction
IO factor	Intracellular accumulation ratio	-	0.83*	0.19	[Z9] p 1332	Volunteers & aids patients (n=40)
GR factor	Granuloma penetration ratio	-	1.25		[Z3] Fig 3	Rabbit lung lesions (n=25)<br>Based on 0.25/0.20

* Note: studies suggest pyrazinamide concentration in ELF will be significantly *higher* than in plasma [Z9], while other studies found it to be significantly *lower* in lesions than plasma [Z3].

Pyrazinamide - Pharmacodynamics

Pyrazinamide is assumed to act independent of any other drugs, and have (a) bactericidal *Bk*, and (b) bacteriostatic effect *Bg*, defined as follows

Bk = 1/(1+(EC50k/C)^ak)) * k(t), for time t, which is added to the effect of other drugs

Bg = (1-1 / (1 + (EC50g/C)^ag)), which is multiplied with the effect from any other drugs

Since pyrazinamide is believed to have no or minimal effect on fast growing bacteria, k(t) is set to 0 for initial 2 days (t) of drug therapy, as detailed further below.

Param	Description	UOM	Value	CV	Source	Population & comments
EC50k	EC50 for bacterial killing	mg/L	100***	-	Estimate based on MIC	
EC50g	EC50 for growth inhibition	mg/L	50***	-	Estimate based on MIC	
MIC	Minimum inhibitory concentration	mg/L	50<br>100<br>200<br>200	-<br>-<br>-	[Z8] p 1003<br>[Z8] p 1003<br>[Z8] p 1003<br>[Z13]	pH 5.5 and 5.7<br>pH 5.8<br>pH 5.95
MIC ratio	Intra- vs. extra-cellular MIC ratio	-	TBD			
αk	Hill curve factor (bactericidal)	-	1.21		[Z6] p 3200	HFS
αg	Hill curve factor (bacteriostatic)	-	1.21		[Z6] p 3200	HFS
Emax extracell	Max kill rate (log10)	CFU/ ml/ day	(0.06)<br>0.11<br>0.10<br>(0.7)<br>0.12<br>0.14<br>**0.12****	-<br>-<br>-<br>-<br>0.25<br>-<br>**0.25**	[Z7] p 1528<br>[Z7] p 1530<br>[Z6] Fig 2<br>[Z13] Fig 2<br>[Z14] Table 1<br>[Z15] Fig 2	Mouse, 300mg/kg, 28 days<br>Guinea pig, 600mg/kg H.E.<br>HFS, 2.89 log10 in 28 days<br>In vitro macrophages<br>HFS and other data<br>In vitro liquid culture<br>Highest/lowest excluded
Emax intracell	Max kill rate (log10)	CFU/ ml/ day			Assumed to be same as for extra-cellular	
$kill_e$	Max kill rate for extracell. (Emax)	1/h	0.01-<br>0.02**			Transformed by ln(10)/24<br>**d0d2d15:**<br>**0.0> 0.02>0.02***

$kill_i$	Max kill rate for intracell. (Emax)	1/h	0.02-0.04**		Assumed to be higher than $kill_e$	Transformed by ln(10)/24 **d0d2d15: 0.0>0.04>0.04***
mutation rate	Mutation rate for mono-resistance	1/gen	1e-6<br>1e-5<br>**1.1e-5**	-<br>-	[Z7] p 1531<br>[Z12] p 5187	Mice / Guinea pig<br>In vitro experiment
mutation rate	Mutation rate for mono-resistance	1/h	4.6e-7			Divide per-generation rate by 24 hr (est. growth cycle)

* Note: pyrazinamide found to have little or no early bactericidal effect, but primarily work on persisting bacteria at low pH [Z9] and [Z16]

** Likely underestimated since based on high dose, and not derived from Emax model

*** EC50 and kill values are highly dependent on current pH conditions, range 0.2x to 2x

Pyrazinamide - References

[Z1] Tostmann, A., et al. (2013) Pharmacokinetics of first-line tuberculosis drugs in Tanzanian patients. *Antimicrob Agents Chemotherapy.* 57(7), 3208-3213. PMID: 23629715

[Z2] Magis-Escurra, C., et al. (2014). Population pharmacokinetics and limited sampling strategy for first-line tuberculosis drugs and moxifloxacin. *Int J Antimicrob Agents.* pii: S0924-8579(14)00164-2. PMID: 24985091

[Z3] Kjelsson, M., et al. (2012). Pharmacokinetic evaluation of the penetration of antituberculosis agents in rabbit pulmonary lesions. *Antimicrobial Agents and Chemotherapy*, 56(1), 446-457. PMID: 21986820

[Z4] Peloquin CA, et al. 1997. Population pharmacokinetic modeling of isoniazid, rifampin, and pyrazinamide. *Antimicrob. Agents Chemother.* 41, 2670-2679. PMID: 9420037

[Z5] Perelman, D. et al. (2004). The Clinical Pharmacokinetics of Pyrazinamide in HIV-Infected Persons with Tuberculosis. *Clinical Infectious Diseases*, 38(4), 556-564. PMID: 14765350

[Z6] Gumbo, T. et al. (2009). Pharmacokinetics-pharmacodynamics of pyrazinamide in a novel in vitro model of tuberculosis for sterilizing effect: a paradigm for faster assessment of new antituberculosis drugs. *Antimicrobial Agents and Chemotherapy.* 53(8), 3197-3204. PMID: 1945130

[Z7] Ahmad et al. (2011). Dose-dependent activity of pyrazinamide in animal models of intracellular and extracellular tuberculosis infections. *Antimicrob Agents Chemother*, 1527–1532. PMID: 21282447

[Z8] Salfinger et al. (1988). Determination of pyrazinamide MICs for *Mycobacterium tuberculosis* at different pHs by the radiometric method. *Antimicrob Agents Chemother.* 32(7), 1002–1004. PMID: 3142340

[Z9] Conte, J., et al. (1999). Intrapulmonary concentrations of pyrazinamide. *Antimicrob Agents Chemother.* 43(6), 1329–1333. PMID: 10348747

[Z10] Grosset, J, et al. (2012). Modeling early bactericidal activity in murine tuberculosis provides insights into the activity of isoniazid and pyrazinamide. *Proc Natl Acad Sci U S A*. 109(37), 15001–15005. PMID: 22927424
[Z11] Zhang, Y., et al. (2003). Mode of action of pyrazinamide: disruption of *Mycobacterium tuberculosis* membrane transport and energetics by pyrazinoic acid. *Journal of Antimicrobial* Chemotherapy. 52, 790–795. PMID: 14563891
[Z12] Stoffels, K., et al. (2012). Systematic analysis of pyrazinamide-resistant spontaneous mutants and clinical isolates of *Mycobacterium tuberculosis*. *Antimicrob. Agents Chemother.* 56(10), 5186-5193. PMID: 22825123
[Z13] Simoes, M. (2009). Lipophilic pyrazinoic acid amide and ester prodrugs stability, activation and activity against M. tuberculosis. *European Journal of Pharmaceutical Sciences* 37(3-4), 257–263. PMID: 19491013
[Z14] Pasipanodya JG, et al. (2011). An oracle: antituberculosis pharmacokinetics-pharmacodynamics, clinical correlation, and clinical trial simulations to predict the future. *Antimicrob. Agents Chemother.* 55, 24–34. PMID: 2093777
[Z15] Hu, Y. et al. (2006). Sterilising action of pyrazinamide in models of dormant and rifampicin-tolerant *Mycobacterium tuberculosis*. *The International Journal of Tuberculosis and Lung Disease.* 10(3), 317–322. PMID: 16562713
[Z16] Zhang, Y. & Mitchison, D. (2003). The curious characteristics of pyrazinamide: a review. Int J *Tuberc Lung Dis*. 7(1), 6-21. PMID: 12701830

Ethambutol

Introduction

Ethambutol is believed to be mainly bacteriostatic. The drug changes the structure of the mycobacterial cell wall, making it more permeable to the other antitubercular drugs and thus increase their effect. It has an elimination half-life of 3-4 hours in healthy patients, which is increased in patients with renal impairment. Approximately 15% of the drug is metabolized in the liver, and it is eliminated mostly by excretion of the unchanged drug in urine.

Ethambutol - Pharmacokinetic model and parameters

Model based on a single-compartment system with first-order absorption and elimination.

Param	Description	UOM	Value	CV	Source	Population & comments
K_a	Coefficient of absorption (oral)	1/h	0.474<br>0.573<br>0.68<br>0.54<br><br>**0.57**	0.39<br>0.17<br>-<br>**-**<br><br>**0.28**	[E3] Table 2<br>[E4] Table 3<br>[E5] Table 2<br>[E6] Table 2	SA TB patients (n=189)<br>Adult volunteers (n=18)<br>Adult TB patients (n=56)<br>Healthy volunteers, fasting (n=14)
CL/F	Total clearance (single dose)<br><br>[Note: per [E3] average for various studies is approx 1.0 L/h*kg, i.e., 65 L/h for 65 kg mass]	L/h	52<br>54.3<br>46.5<br>47.6<br><br>80.5<br>68.4<br><br>78.2<br>**61.1**	-<br><br>0.27<br>0.20<br><br>-<br>0.20<br><br>0.27<br>**0.24**	[E1] Table 2<br>[E2] Table 2<br>[E13] Tab 2<br>[E3] Table 3<br><br>[E5] Table 5<br>[E6] Table 2<br><br>[E4] Table 2	Tanzania TB patients (n=20)<br>Dutch TB patients (n=19)<br>Dutch NTB patients (n=14)<br>SA TB patients (n=189), scaled to 60 kg<br>Adult TB patients (n=56)<br>Healthy volunteers, fasting (n=14), scaled to 60 kg<br>Adult volunteers (n=18)
V/F	Effective volume of distribution	L	719<br>723<br>672<br>(99)<br><br>382<br>446<br>(232)<br><br>**230**	-<br><br>0.08<br>0.42<br><br>-<br>0.58<br>0.51<br><br>**0.33**	[E1] Table 2<br>[E2] Table 2<br>[E13] Tab 2<br>[E3] Table 3<br><br>[E5] Table 2<br>[E4] Table 2<br>[E6] Table 2	Tanzania TB patients (n=20)<br>Dutch TB patients (n=19)<br>Dutch NTB patients (n=14)<br>SA TB patients (n=189), scaled to 60 kg<br>Adult TB patients (n=56)<br>Adult volunteers (n=18)<br>Healthy volunteers, fasting (n=14), scaled to 60 kg<br>Selected estimates
Ke (CL/V)	Coefficient of elimination (total)	1/h	0.072<br>0.075<br>0.069<br>(0.48)	 <br>-<br>0.42	[E1]<br>[E2]<br>[E13]<br>[E3]	

			0.175<br>0.211<br>(0.29)<br>0.17<br>**0.129**	0.23<br>-<br>0.51<br>0.36<br>**0.30**	[E4]<br>[E5]<br>[E6]<br>[E7]	Adult volunteers (n=18)<br><br>TB patients, HIV+ (n=31)<br><br>Excluding 2 highest values
K_e mult	Auto-induction multiplier	-	1.0	**-**		No auto induction
K_e time	Auto-induction time	days	-	**-**		No auto induction
IO factor	Intracellular accumulation ratio	-	7<br>**1.5**	**-**	[E12] abstr.	Method unknown<br>Selected estimate
GR factor	Granuloma penetration ratio	-	0.45	**-**		Selected estimate

Ethambutol - Pharmacodynamics

Individual drugs assumed to act independently with a) bactericidal *Bk*, and b) bacteriostatic effect *Bg*, where

Bk = 1/(1+(EC50k/C)^ak)) * k(t), for time t, in a given model compartment, added for N drugs
Bg = (1-1 / (1 + (EC50g/C)^ag)), multiplied across N drugs

It is believed ethambutol can have synergistic effect with rifampin and isoniazid, due to the ability of ethambutol to reduce integrity of the bacterial cell wall to allow increased concentration of RIF and INH to enter the bacteria [E8]. The current simulation model does <u>not</u> include synergistic effects.

Param	Description	UOM	Value	CV	Source	Population & comments
$EC50_k$	EC50 for bacterial killing	mg/L	5.38<br>**4.0**	0.16	[E11] p 1729	HFS, Note: *M. avium*<br>Selected estimate
$EC50_g$	EC50 for growth inhibition	mg/L	**1.0**			Selected estimate
MIC	Minimum inhibitory concentration	mg/L	(8)<br><br>0.03	-<br><br>-	[E15] Fig 1<br><br>[E9]	Mouse macrophages, in vitro<br>HFS
MIC ratio	Intra- vs. extra-cellular MIC ratio	-				
α_k	Hill curve factor (bactericidal)	-	1.0			Selected estimate

α_g	Hill curve factor (bacteriostatic)	-	1.0			Selected estimate
Emax extracell	Max kill rate	CFU/ ml/ day	0.22<br>0.26<br>(0.16)<br>0.7<br>(0.79)<br>**0.40**	0.17<br>-<br>-<br><br>0.07<br>**0.17**	[E9] p 1228<br>[E8] p 29<br>[E8] p 29<br>[E10] Figure 2<br>[E11] p 1729	HFS, (0.04-0.1 after day 2)<br>First 2 days<br>Days 3-14<br>In vitro, (2.2 after 3 days)<br>HFS, Note: M. avium<br>Skip highest/lowest values
Emax intracell	Max kill rate	CFU/ ml/ day	same as above	-		
$kill_e$	Max kill rate per hour in base **e**	1/h	0.04	-		Transformed by ln(10)/24<br>**d0d2d15: 0.05>0.04>0.03***
$kill_i$	Max kill rate per hour in base **e**	1/h	0.04	-		Transformed by ln(10)/24<br>**d0d2d15: 0.05>0.04>0.03***
mutation rate	Mutation rate for mono-resistance	1/gen	1.57e-5<br>1e-7<br>7.25e-7<br>**5.5e-6**	-	[E9] Page 4<br>[E14] Resist.<br>[E11] p 1729	HFS<br><br>HFS, Note: *M. avium*
mutation rate	Hourly mutation rate	1/h	**2.3e-7**	-		Divide per-generation rate by 24 hr (est. growth cycle)

* Kill rate believed to decline significantly after initial 1-2 weeks of drug therapy [E8]

Ethambutol - References

[E1] Tostmann, A., et al. (2013) Pharmacokinetics of first-line tuberculosis drugs in Tanzanian patients. *Antimicrob Agents Chemotherapy.* 57(7), 3208-3213. PMID: 23629715

[E2] Magis-Escurra, C., et al. (2014). Population pharmacokinetics and limited sampling strategy for first-line tuberculosis drugs and moxifloxacin. *Int J Antimicrob Agents.* pii: S0924-8579(14)00164-2. PMID: 24985091

[E3] Jönsson, S., et al. (2011). Population pharmacokinetics of ethambutol in South African tuberculosis patients. *Antimicrob Agents Chemother*. 55(9), 4230-4237. PMID: 21690284

[E4] Hall, R., et al. (2011). Ethambutol pharmacokinetic variability is linked to body mass in overweight, obese, and extremely obese people. *Antimicrob Agents Chemother.* 56(3), 1502-7. PMID: 22155817

[E5] Zhu, M., et al. (2004). Pharmacokinetics of ethambutol in children and adults with tuberculosis. *Int J Tuberculosis Lung Dis*. 8(11), 1360–1367. PMID: 15581206

[E6] Peloquin, C. et al. (1999). Pharmacokinetics of ethambutol under fasting conditions, with food, and with antacids. *Antimicrob Agents Chemother.* 43(3), 568–572. PMID: 10049268

[E7] Perlman, D. et al. (2005). The clinical pharmacokinetics of rifampin and ethambutol in HIV-infected persons with tuberculosis. *Clin Infect Dis*, 41(11), 1638-1647. PMID: 16267738
[E8] Pasipanodya JG, et al. (2011). An oracle: antituberculosis pharmacokinetics-pharmacodynamics, clinical correlation, and clinical trial simulations to predict the future. *Antimicrob. Agents Chemother.* 55, 24–34. PMID: 2093777
[E9] Srivastava, S., et al. (2010). Efflux-pump-derived multiple drug resistance to ethambutol monotherapy in *Mycobacterium tuberculosis* and the pharmacokinetics and pharmacodynamics of ethambutol. *J. Infect. Dis.* 201**,** 1225-1231. PMID: 20210628
[E10] Steenwinkel, J., et al. (2010). Time-kill kinetics of anti-tuberculosis drugs, and emergence of resistance, in relation to metabolic activity of *Mycobacterium tuberculosis*. *J Antimicrob Chemother.*, 65(12), 2582-2589. PMID: 20947621
[E11] Deshpande, D., et al. (2010). Ethambutol optimal clinical dose and susceptibility breakpoint identification by use of a novel pharmacokinetic-pharmacodynamic model of disseminated intracellular *Mycobacterium avium*. *Antimicrob Agents Chemother.* 54(5), 1728-33. PMID: 20231389
[E12] Johnson J., et al. (1980). Antibiotic uptake by alveolar macrophages. *J Lab Clin Med.* 95(3), 429-39. PMID: 7354244
[E13] Magis-Escurra, C. et al. (2013). Pharmacokinetic studies in patients with nontuberculous mycobacterial lung infections. *Int J Antimicrob Agents.* 42(3), 256-61. PMID: 23837923
[E14] Drew, R. et al. Ethambutol. In: UpToDate, Post TW (Ed), UpToDate, Waltham, MA. (Accessed on September 3, 2014.)
[E15] Jhamb S. et al. (2014). Determination of the activity of standard anti-tuberculosis drugs against intramacrophage *Mycobacterium tuberculosis*, in vitro: MGIT 960 as a viable alternative for BACTEC 460. *Braz J Infect Dis.* 18(3), 336-40. PMID: 24709416
[E16] Chimote, G. & Banerjee, R. (2008). Evaluation of antitubercular drug insertion into preformed dipalmitoylphosphatidylcholine monolayers. *Colloids Surf B Biointerfaces.* 62(2), 258-64. PMID: 18082382
[E17] Liss, (1982). Bactericidal activity of ethambutol against extracellular *Mycobacterium tuberculosis* and bacilli phagocytized by human alveolar macrophages. *S Afr Med J.*, Spec No:15-9. PMID: 6813978
